# Inside‐Outside ROS Therapeutic Strategy Based on Piezoelectric Nano‐Urchin for Drug‐Resistant Bacteria Biofilm Infections

**DOI:** 10.1002/advs.76086

**Published:** 2026-06-12

**Authors:** Xinjian Guo, Jin Yang, Mengjie An, Bingjie Lin, Tao Liu, Limin Zhang

**Affiliations:** ^1^ Shanghai Key Laboratory of Green Chemistry and Chemical Processes School of Chemistry and Molecular Engineering East China Normal University Shanghai P. R. China; ^2^ Institute of Cardiovascular Translational Medicine Putuo Hospital Shanghai University of Traditional Chinese Medicine Shanghai P. R. China

**Keywords:** biofilms, drug‐resistant bacteria, piezoelectric nanozymes, ROS

## Abstract

Biofilm‐associated infections pose a critical clinical challenge due to their inherent antibiotic resistance and limited therapeutic penetrability. Herein, we engineered a mechano‐piezoelectric nano‐urchin system, NiCo_2_S_4_@UiO‐66, which utilizes ultrasound to achieve mechanical biofilm disruption and spatially hierarchical reactive oxygen species (ROS) generation for synergistic antimicrobial therapy. The spiky architecture of NiCo_2_S_4_ nano‐urchins acts as physical penetrators, mechanically compromising biofilm integrity. Under ultrasound activation, a graded ROS generation mechanism is greatly enhanced via two distinct pathways. Externally, the NiCo_2_S_4_ nanozyme activated by piezoelectric UiO‐66 successfully catalyzes pathogenic H_2_O_2_ at the biofilm periphery into highly destructive ·OH radicals, which not only degrade the extracellular polymeric matrix, but avoids additional oxidative stress. Internally, the mechanically driven piezoelectric UiO‐66 component generates long‐diffusing singlet oxygen (^1^O_2_), capable of targeting and eliminating bacteria embedded deep within the biofilm. Driven by the nano‐urchin mechanical action, this hierarchical ROS mechanism integrates intra‐biofilm ^1^O_2_ production with peripheral ·OH‐mediated decomposition, ensuring robust and comprehensive biofilm eradication. In a murine model of methicillin‐resistant *Staphylococcus aureus* (*MRSA*) infected wounds, the system achieved rapid biofilm clearance and accelerated tissue repair through immunomodulation and angiogenesis promotion. This strategy addresses key limitations of conventional antimicrobial therapies and offers an effective approach for treating multidrug‐resistant biofilm infections.

## Graphical abstract

The contribution of this study lies not only in enabling effective treatment of drug‐resistant biofilm infections, but also in establishing a transferable therapeutic paradigm: Ultrasound‐triggered mechanical forces synergistically enhance piezoelectric polarization and nanozyme activity, driving functional ROS generation inside and outside biofilms, thereby overcoming biofilm barriers and antimicrobial resistance.

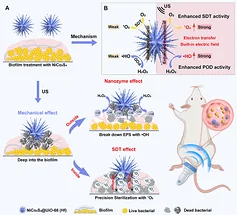

## 1 Introduction

Antibiotic‐resistant bacteria continue to emerge and spread globally, evolving into a systemic public health crisis that contributes to nearly one million deaths annually from antimicrobial‐resistant infections [1, 2]. Multidrug‐resistant bacteria not only increase morbidity and mortality among infected individuals but also negatively impact clinical outcomes across diverse patient populations [3]. One of the core factors underlying the resilience of resistant bacteria is biofilm formation, which poses a major therapeutic barrier in clinical settings [4, 5, 6]. As a key defense strategy employed by resistant pathogens, biofilms construct highly organized three‐dimensional communities encased within self‐produced extracellular polymeric substances (EPS) [7, 8]. These “microbial cities” not only hinder antibiotic penetration but also reinforce bacterial tolerance through multiple synergistic mechanisms. Furthermore, the heterogeneous microenvironment within mature biofilms promotes the selection and proliferation of persistent resistant subpopulations, often leading to recurrent infections and substantially complicating treatment efforts [9, 10]. Consequently, developing novel therapeutic strategies capable of penetrating and disrupting this sophisticated biofilm architecture represents a critical challenge in the fight against antimicrobial resistance.

ROS‐based strategies represent promising approaches against drug‐resistant infections, benefiting from their nonspecific oxidative mechanisms that circumvent conventional resistance pathways and induce substantial damage to biomacromolecules, thereby conferring broad‐spectrum antimicrobial activity [11, 12, 13]. In recent decades, considerable efforts have been devoted to developing nanocomposites that introduce defects or construct heterostructures to regulate energy band structures, and facilitate the separation of electron–hole pairs, improving the generation of ROS. For instance, Liu et al. constructed an acoustic Cu‐doped defective TiO_2_ superstructure coating (Cu‐TiO_x_). The redistribution of Cu atoms broke the pristine lattice of TiO_2_ during the thermal reduction treatment, regulating its energy structure and promoting electron–hole pair separation under ultrasound (US) radiation [14]. Luo and Feng et al. developed a donor‐acceptor COF‐based sonozyme loading with Pt nanoparticles, in which the band position was optimized, and Pt nanoparticles improved US‐excited charge separation and transfer dynamics, enhancing the US‐triggered ROS generation [15]. Recently, Li et al. developed Cu single‐atom embedded g‐C_3_N_4_ nanosheets, where nitrogen vacancies significantly enhanced photocatalytic glucose oxidation, and atomically dispersed Cu promoted the generation of ·OH and O_2_^·−^ to efficiently eliminate multidrug‐resistant bacteria [16]. In these systems, multiple ROS species are often simultaneously generated to overwhelm bacterial defenses. However, excessive ROS release may disrupt physiological redox homeostasis and lead to overtreatment [17]. Moreover, different ROS species possess distinct functional properties [18, 19, 20, 21, 22, 23, 24]. For example, hydroxyl radical (·OH) exhibits high reactivity, but extremely short diffusion distance restricting its further action to targets in immediate proximity, making it insufficient for eradicating bacteria deeply embedded within biofilms. In contrast, although singlet oxygen (^1^O_2_) exhibits weaker direct matrix degradation capability, its longer half‐life and deeper diffusion capacity enable it to selectively inactivate bacteria within biofilms by oxidizing lipids and specific amino acid residues [25, 26, 27]. These differences have been recognized to be differentially exploited in antibacterial therapy. For example, Li et al. developed a smart strategy based on a carbon‐dot‐based ROS nano‐modulator capable of selectively scavenging cytotoxic ROS (·OH and ONOO−) while preserving essential antibacterial ROS (O_2_^·−^, H_2_O_2_, and NO) [28]. Thus, the strategy selection of ROS types was necessary and should be carefully considered. Although various modalities, including photodynamic and sonodynamic therapies, have shown potential for biofilm eradication, conventional strategies often overlook the intrinsic functional diversity among ROS species [25, 26, 27]. This oversight inevitably constrains the efficacy and further development of ROS‐based antibacterial systems.

**SCHEME 1:**
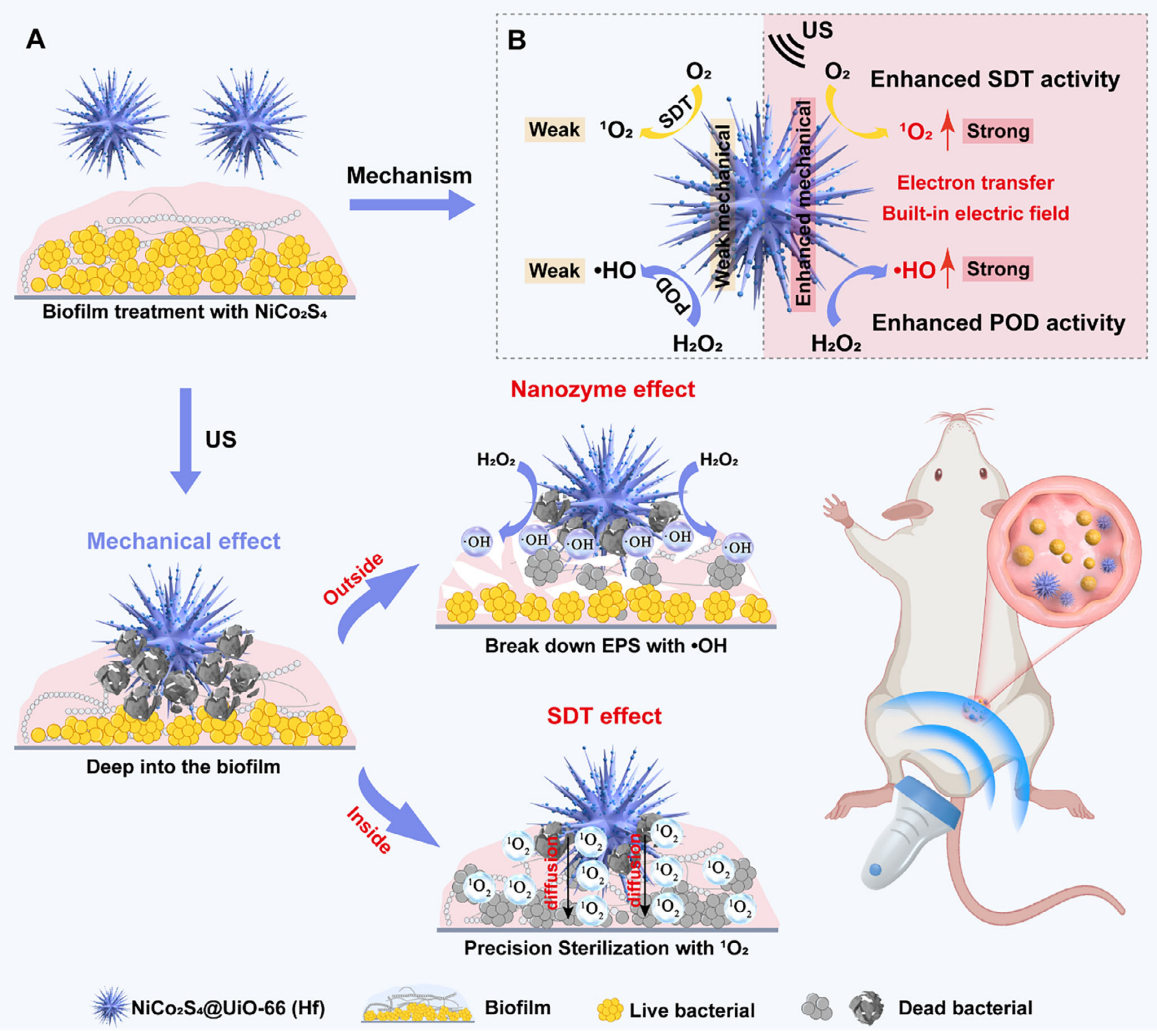
(A) Schematic of spatially hierarchical ROS release by piezoelectric nano‐urchin for treatment of biofilm infections in drug‐resistant bacteria. (B) Schematic of the piezoelectric nanozyme mechanism of NiCo_2_S_4_@UiO‐66.

In this study, we engineer a mechano‐piezoelectric nano‐urchin system, NiCo_2_S_4_@UiO‐66, which utilizes US to achieve mechanical biofilm disruption based on functional diversity among ROS species for synergistic antimicrobial therapy (Scheme 1). The spiky architecture of NiCo_2_S_4_ nano‐urchins acts as physical penetrators, mechanically compromising biofilm integrity. Under US activation, the ^1^O_2_ and ·OH generation mechanism is rationally enhanced through two distinct pathways. Externally, the spiky NiCo_2_S_4_ aided by the piezoelectric activity of UiO‐66, exhibits significantly enhanced nanozyme activity with a low Michaelis–Menten constant (*K*_m_ (0.64 mM)) and high *V*_max_ (15.06 ×10^−8^ M s^−1^). It efficiently catalyzes pathogenic H_2_O_2_ present at the biofilm periphery into highly destructive ·OH radicals, which effectively degrade the extracellular polymeric matrix. Internally, the mechanically driven piezoelectric UiO‐66 component generates long‐diffusing singlet oxygen (^1^O_2_), capable of targeting and eliminating bacteria embedded deep within the biofilm. Driven by the nano‐urchin mechanical action, this hierarchical ROS mechanism integrates intra‐biofilm ^1^O_2_ production with peripheral ·OH‐mediated decomposition, ensuring robust and comprehensive biofilm eradication. In a murine model of methicillin‐resistant *Staphylococcus aureus* (*MRSA*)‐infected wounds, the system not only effectively clears biofilms and bacteria but also promotes tissue repair through inflammation modulation and enhanced angiogenesis.

## 2 Results and Discussion

### 2.1 Synthesis and Characterization of Urchin‐Like NiCo_2_S_4_@UiO‐66

**FIGURE 1:**
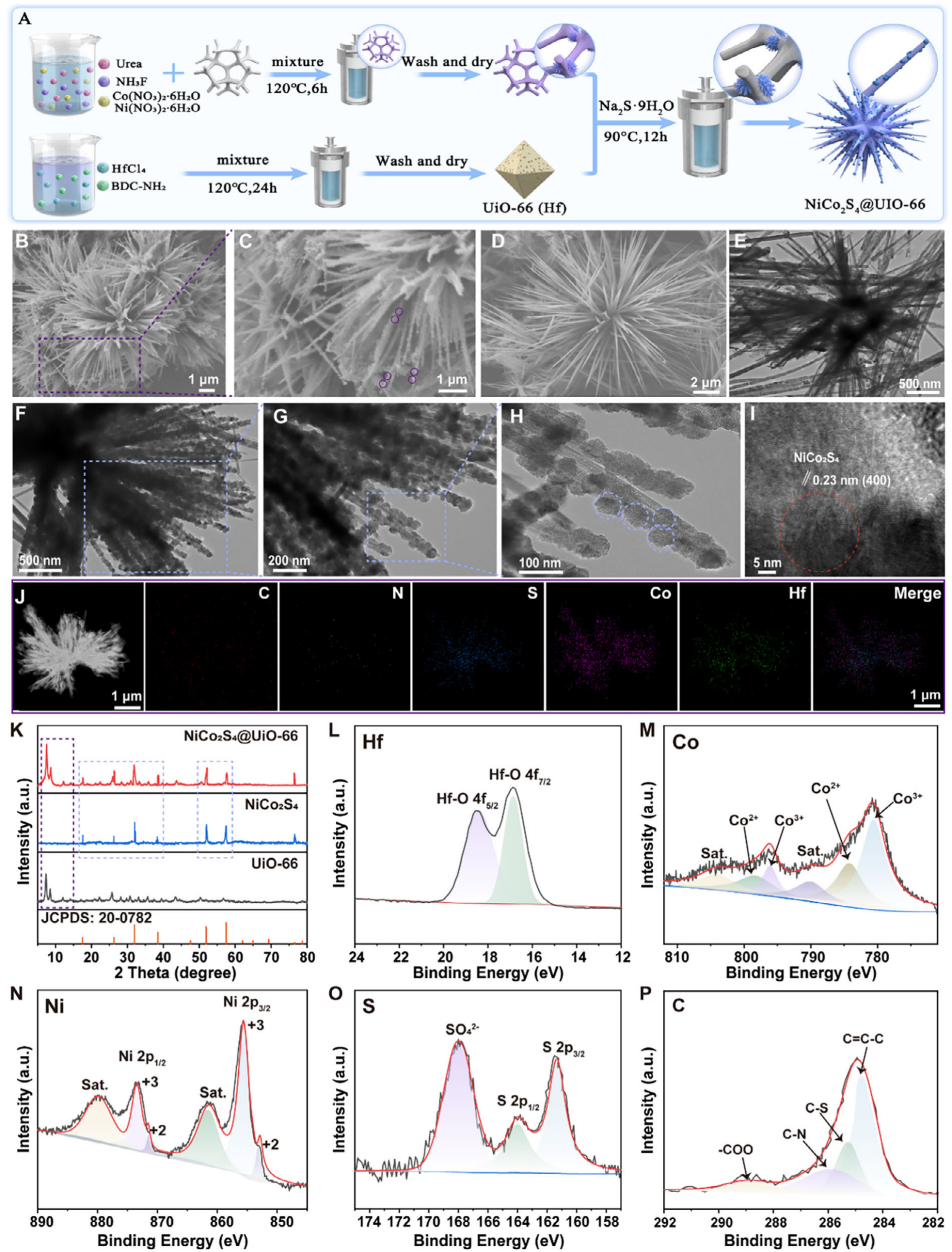
(A) Schematic of the NiCo_2_S_4_@UiO‐66 synthesis. (B,C) SEM images of NiCo_2_S_4_@UiO‐66. (D) SEM image of NiCo_2_S_4_. (E) TEM images of NiCo_2_S_4_. (F–H) TEM images of NiCo_2_S_4_@UiO‐66. (I) HR‐TEM image of NiCo_2_S_4_@UiO‐66. (J) EDX elemental distribution map of NiCo_2_S_4_@UiO‐66. (K) PXRD patterns of UiO‐66, NiCo_2_S_4_, and NiCo_2_S_4_@UiO‐66. High‐resolution XPS spectra of (L) Hf, (M) Co, (N) Ni, (O) S, and (P) C elements at NiCo_2_S_4_@UiO‐66.

The urchin‐like NiCo_2_S_4_@UiO‐66 heterostructure was synthesized via a two‐step process as illustrated in Figure 1A. First, a hydrothermal reaction involving Co(NO_3_)_2_, Ni(NO_3_)_2,_ and urea as a pH regulator was employed to grow Ni‐Co(OH)_2_ precursor on a nickel foam substrate. This was followed by a hydrothermal sulfidation step, in which the as‐prepared piezoelectric UiO‐66 (Hf) (Figure S1) was introduced together with the freshly synthesized Ni‐Co(OH)_2_ to directly form UiO‐66‐decorated NiCo_2_S_4_, denoted as NiCo_2_S_4_@UiO‐66 (Figure 1A). Scanning electron microscopy (SEM) images revealed that the resulting NiCo_2_S_4_@UiO‐66 preserves an urchin‐like nanoarchitecture resembling that of pristine NiCo_2_S_4_, comprising elongated spikes several micrometers in length (Figure 1B–D and Figure S2). TEM analysis showed numerous small nanoparticles distributed across the framework, assembled in a bead‐on‐a‐string morphology (Figure 1E–H). High‐resolution TEM imaging revealed well‐defined lattice fringes with a spacing of 0.23 nm, corresponding to the (400) plane of the spiky NiCo_2_S_4_ (Figure 1J). Elemental mapping via energy‐dispersive X‐ray spectroscopy (EDX) confirmed the uniform distribution of Hf, Ni, Co, and S throughout the NiCo_2_S_4_@UiO‐66 heterostructure (Figure 1I). X‐ray diffraction (XRD) confirmed that the characteristic peaks of both UiO‐66 and NiCo_2_S_4_ appear at the same positions in NiCo_2_S_4_@UiO‐66, in which the peaks at 16.4°, 27.1°, 32.3°, 38.6°, 51.8°, and 57.5° 2θ correspond to the (111), (220), (311), (400), (422), (511), and (440) planes of NiCo_2_S_4_, while the peaks at 4.8° and 7.2° 2θ are assigned to the (111) and (200) planes of UiO‐66 (Figure 1K). These observations indicated that UiO‐66 doping did not alter the original urchin‐like morphology of NiCo_2_S_4_.

X‐ray electron spectroscopy (XPS) analysis was performed to elucidate the chemical states of UiO‐66 dopants on NiCo_2_S_4_. The Hf 4f spectrum exhibited two peaks at 16.9 and 18.5 eV characteristic of Hf ^4+^ (Figure 1L). In the high‐resolution Co 2p spectrum of NiCo_2_S_4_@UiO‐66, two spin‐orbit double peaks corresponding to Co 2p^3/2^ and Co 2p^1/2^ along with satellite feature were identified. Peaks observed at 798.40 and 784.54 eV are assigned to Co^2+^, while those at 796.23 and 780.76 eV originated from Co^3+^ (Figure 1M). The Ni 2p region was fitted with two spin‐orbit doublets and two shark‐up satellites, where the peaks at 853.2 and 870.5 eV are attributed to Ni^2+^, and those at 855.9 and 873.8 eV to Ni^3+^ (Figure 1N). S 2p spectrum (Figure 1O) displays a peak at 161.3 eV, indicative of S^2−^, along S 2p^1/2^ component at 163.9 eV ascribed to Ni‐S and Co‐S bonds, and another at 168.1 eV for sulfur oxides. In the C 1s spectrum, the main peak at 284.8 eV was assigned to C═C─C bonds, while peaks at 285.2 and 285.9 eV correspond to C─S and C─N bonds, respectively. These data confirmed that NiCo_2_S_4_@UiO‐66 was successfully synthesized, and the UiO‐66 carbon was chemically integrated with the NiCo_2_S_4_ framework (Figure 1P). In addition, zeta potential measurements yielded a value of 13.5 mV for NiCo_2_S_4_@UiO‐66, indicating its positively charged feature (Figure S3). Moreover, NiCo_2_S_4_@UiO‐66 composites from three independently synthesized batches were characterized. All batches retained the typical sea urchin‐like morphology without structural collapse morphological deviation or structural collapse, and similar zeta potentials (Figure S4A,B). The loading content of UiO‐66 in the NiCo_2_S_4_@UiO‐66 system was remained at ∼28.7%, with small deviations of 7.6% among samples and 8.4% across batches, indicating good batch‐to‐batch reproducibility of NiCo_2_S_4_@UiO‐66 (Figure S4C). In addition, we tracked morphological changes of NiCo_2_S_4_@UiO‐66 after prolonged dispersion in PBS for 15 days, as well as both before and after US treatment. TEM results showed that the morphology remained stable after 15 days in PBS, with no evident deterioration or structural damage (Figure S4D). After US treatment, the overall structure of NiCo_2_S_4_@UiO‐66 maintained its integrity, with only minor broken spines and no obvious framework collapse (Figure S4E). These results confirmed that the NiCo_2_S_4_@UiO‐66 composite possessed good reproducibility and structural stability under the US conditions.

### 2.2 Performance Evaluation of NiCo_2_S_4_@UiO‐66

**FIGURE 2:**
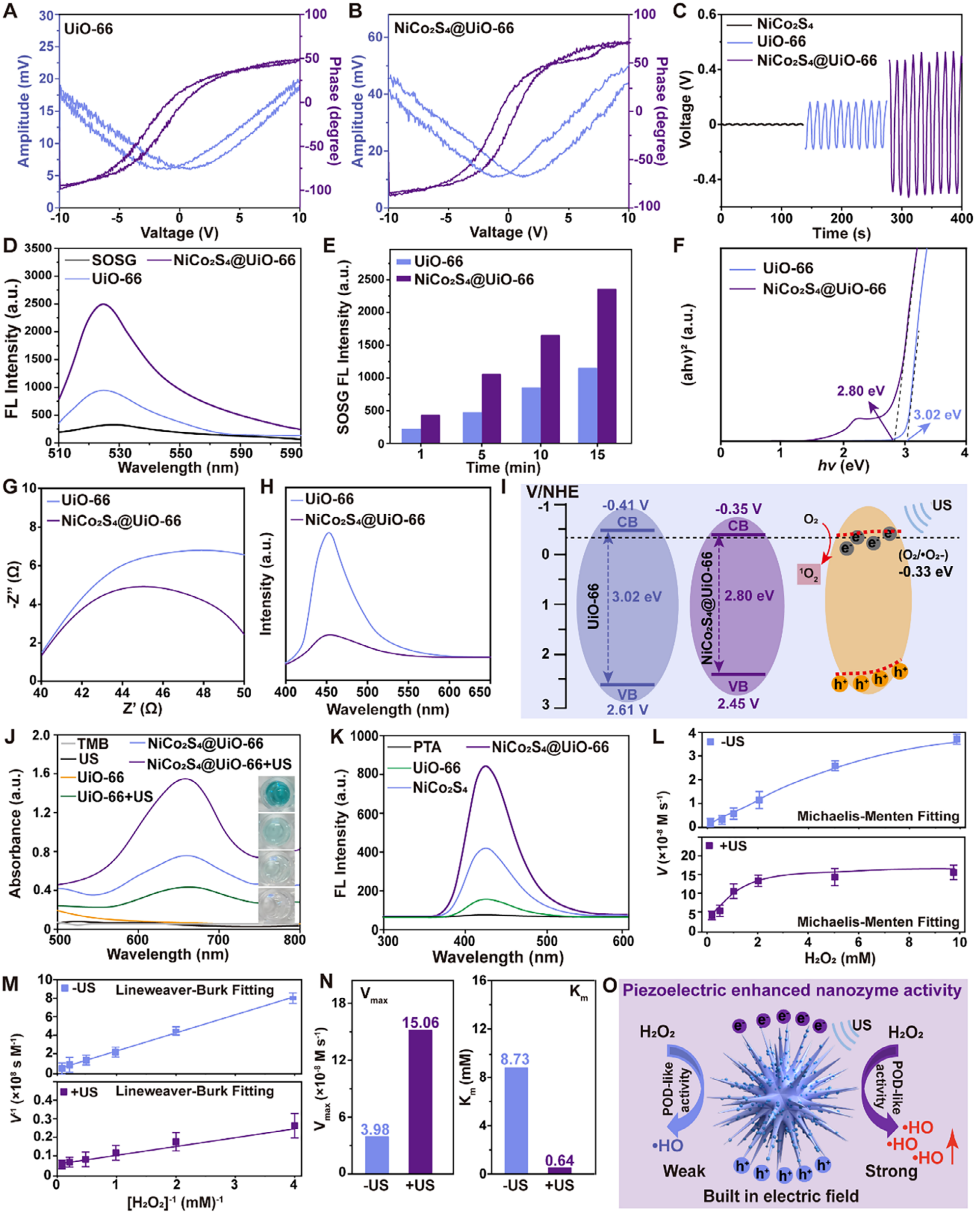
Phase hysteresis loop and butterfly hysteresis loop of (A) UiO‐66 and (B) NiCo_2_S_4_@UiO‐66. (C) Voltage produced by UiO‐66 and NiCo_2_S_4_@UiO‐66 under ultrasound irradiation. (D) Fluorescence spectrum of the catalyzed oxidation of SOSG. (E) Fluorescence intensity of the catalyzed oxidation of SOSG in different component conditions. (F) Tauc plots of UiO‐66 and NiCo_2_S_4_@UiO‐66 under ultrasound irradiation. (G) Nyquist plots, (H) PL spectra of UiO‐66 and NiCo_2_S_4_@UiO‐66 under US irradiation. (I) Proposed mechanism for the enhancement of piezo‐enhanced SDT on the NiCo_2_S_4_@UiO‐66. (J) UV–vis intensity changes of oxTMB by POD‐like activity produced by different components under the same US irradiation time. (K) Fluorescence spectra of the catalyzed oxidation of PTA with different components of NiCo_2_S_4_@UiO‐66. (L) Michaelis‐Menten kinetic analysis, and (M) Lineweaver‐Burk plotting with H_2_O_2_ as the substrate. (N) Kinetic parameters of POD‐like activity. (O) Proposed mechanism for piezoelectric‐enhanced nanozyme activity on the NiCo_2_S_4_@UiO‐66.

The piezoelectric properties of NiCo_2_S_4_@UiO‐66 were assessed by piezoelectric force microscopy (Figure 2A,B). Both UiO‐66 and NiCo_2_S_4_@UiO‐66 displayed typical butterfly curves and phase hysteresis upon varying the electric field from −10 to 10 V. Compared to UiO‐66, NiCo_2_S_4_@UiO‐66 exhibited a larger amplitude in the butterfly loop, in which the *d*_33_ value of NiCo_2_S_4_@UiO‐66 was estimated 134 pm V^−1^ larger than that of UiO‐66 (68 pm V^−1^). Moreover, a larger peak voltage of 462 mV was obtained at NiCo_2_S_4_@UiO‐66 than UiO‐66 (196 mV), while no piezoelectric signal was observed at NiCo_2_S_4_ alone (Figure 2C). These results indicated that the incorporation of NiCo_2_S_4_ efficiently increased the imbalance in the charge distribution on the NiCo_2_S_4_@UiO‐66 surface, thereby enhancing the piezoelectric performance of UiO‐66, which implies a potential improvement in its sonodynamic therapy (SDT) efficacy. Given previous reports that UiO‐66 could generate singlet oxygen (^1^O_2_) under US stimulation, we used singlet oxygen sensor green (SOSG) as a specific probe to detect ^1^O_2_ [29]. As shown in Figure 2D, the SOSG fluorescence intensity at 525 nm was obviously increased upon US exposure, and the amount of ^1^O_2_ produced by NiCo_2_S_4_@UiO‐66 was nearly twice that generated by UiO‐66 under the same time period (Figure 2E). These results indicated that the composite of NiCo_2_S_4_ apparently enhanced the potential SDT performance of UiO‐66. To further investigate the mechanism underlying the enhanced SDT activity, we examined the band gap structures of NiCo_2_S_4_@UiO‐66 and UiO‐66 using UV–vis diffuse reflectance spectroscopy. Tauc plot analysis indicated that NiCo_2_S_4_@UiO‐66 had a narrower band gap (2.80 eV) than UiO‐66 (3.02 eV) (Figure 2F). Meanwhile, electrochemical impedance spectroscopy showed that NiCo_2_S_4_@UiO‐66 had lower electron transfer resistance (Figure 2G). In addition, photoluminescence (PL) spectroscopy showed a 5.2‐fold reduction in PL intensity, indicating that NiCo_2_S_4_ efficiently suppresses electron–hole recombination in UiO‐66 (Figure 2H). The possible mechanism of the improved piezoelectric SDT effect was shown in Figure 2I. According to VB‐XPS result, the valence band (VB) positions of NiCo_2_S_4_@UiO‐66 and UiO‐66 were estimated to be 2.39 and 2.45 eV (Figure S5). Under US irradiation, the electron–hole pairs migrated to different side of the NiCo_2_S_4_@UiO‐66 composite. Simultaneously, internal polarization occurred, leading to band bending where the band energy increased on the CB and decreased on the VB. Band bending reduced the distance between the CB edge and the redox potential of ROS and promoted the separation of the electron–hole pairs, thereby facilitating the ROS‐generating chemical reactions of the electrons and holes with the surrounding O_2_ molecules. Instead of recombining with the holes, the generated electron transferred to the NiCo_2_S_4_ and reacted directly with O_2_. Both the reduced band gap width and increased piezoelectric coefficient contributed to the improvement in ROS production efficiency.

The NiCo_2_S_4_ component in NiCo_2_S_4_@UiO‐66 still retains peroxidase‐like (POD) activity, enabling it to catalyze the decomposition of H_2_O_2_ into ·OH. Using 3,3',5,5'‐tetramethylbenzidine (TMB) as a chromogenic ROS substrate [30], negligible absorbance change was observed in TMB solution with and without US irradiation (Figure 2J). The addition of UiO‐66 couldn't give rise to apparent absorption, while an obvious absorbance at ∼652 nm was observed upon US irradiation, confirming the successful generation of ROS. Moreover, the intensity at 652 nm was slightly enhanced under US compared to NiCo_2_S_4_ alone (Figure S6A), suggesting that US itself could facilitate the interaction between H_2_O_2_ and NiCo_2_S_4_, improving its enzyme‐like activity. In contrast, larger absorption peaks at ∼652 nm were obtained in NiCo_2_S_4_@UiO‐66 solution, confirming the successful generation of ·OH due to their intrinsic peroxidase‐mimicking property. Such catalytic activity was further enhanced by US irradiation, leading to a stronger absorption at 652 nm. The generation of ·OH was further verified using p‐terephthalic acid (PTA) as a specific fluorescence probe [31]. The fluorescent intensity at 425 nm was both observed for NiCo_2_S_4_ and NiCo_2_S_4_@UiO‐66, indicating these two materials could catalyze H_2_O_2_ to ·OH. By contrast, the same amount of NiCo_2_S_4_@UiO‐66 generated approximately 2.2 times more ·OH than NiCo_2_S_4_ (Figure 2K). Under US irradiation, NiCo_2_S_4_ alone produced a higher fluorescent signal than without US, confirming that US promotes ·OH generation even without a piezoelectric contribution (Figure S6B). This US‐induced fluorescent increase was greatly larger in the NiCo_2_S_4_@UiO‐66 system than in NiCo_2_S_4_ alone. According to previous investigation [32, 33], the US parameters used in our system (0.5 W cm^−2^, at 1 MHz) satisfied the conditions for inertial cavitation. However, the overall cavitation efficiency at this frequency was relatively low, resulting in only a trace amount of radical generation [34]. As a result, the contribution of US‐induced enhancement to the overall enzymatic activity was small, while the piezoelectric effect arising from the NiCo_2_S_4_@UiO‐66 heterointerface played the dominant role.

To quantitatively assess the peroxidase‐like activity with and without US, we performed steady‐state kinetics assays by incubating NiCo_2_S_4_@UiO‐66 with TMB and H_2_O_2_ at concentrations ranging from 1 to 100 mm. Time‐dependent absorbance changes of oxTMB with and without US are shown in Figure S7. The kinetics conformed to Michaelis–Menten model (Figure 2L), and Lineweaver–Burk analysis (Figure 2M) revealed that under US irradiation, NiCo_2_S_4_@UiO‐66 displayed a lower *K*_m_ (0.64 mm) and higher *V*_max_ (15.06 ×10^−8^ M s^−1^) compared to the non‐US conditions (*K*_m_ = 8.73 mM, *V*_max_ = 3.98 ×10^−8^ M s^−1^) (Figure 2N). These findings indicated that US promoted the catalytic interaction between NiCo_2_S_4_@UiO‐66 and H_2_O_2_, significantly enhancing its peroxidase‐like activity (Figure 2O and Figure S8).

**FIGURE 3:**
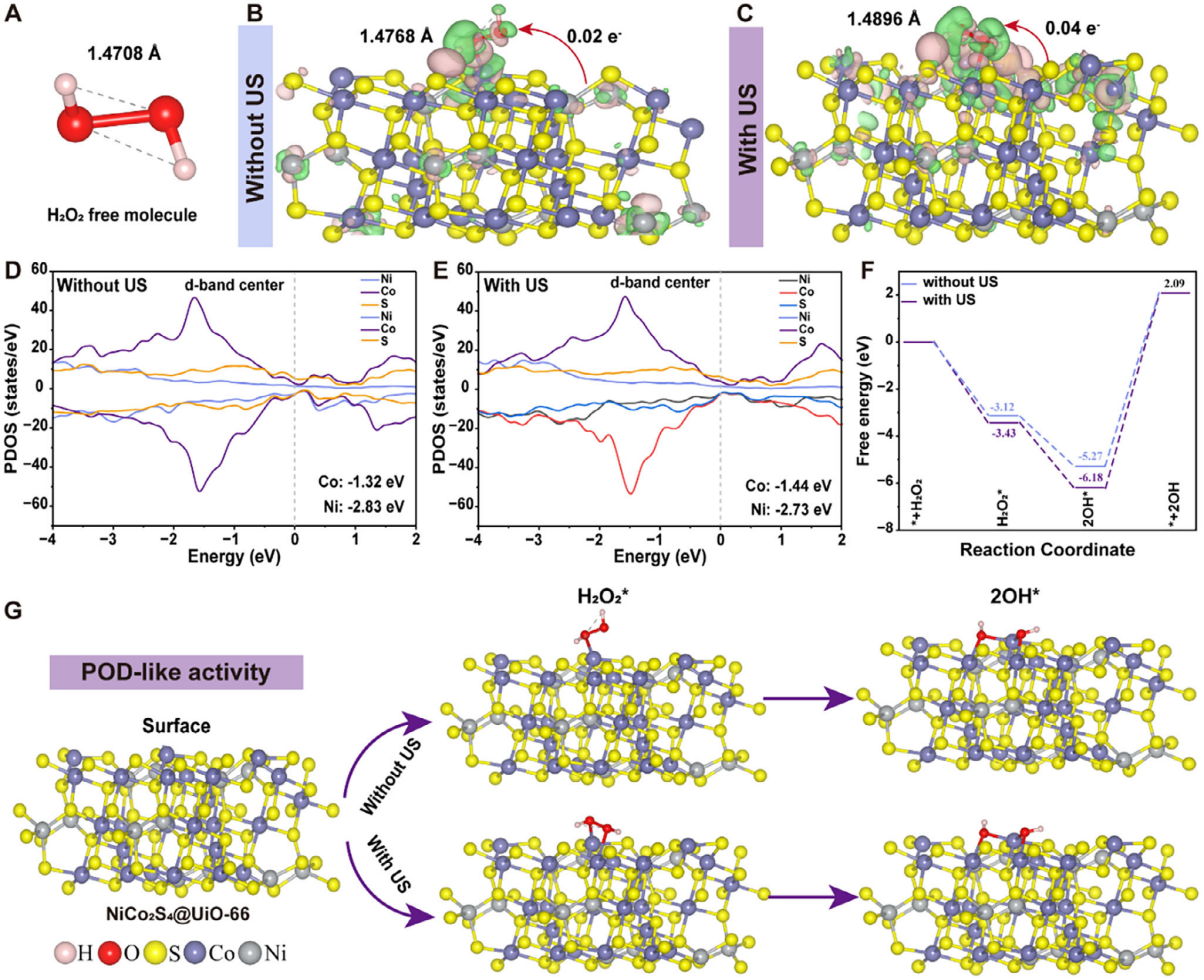
(A) Structural model of the H_2_O_2_ molecule and the corresponding O─O bond length. Differential charge‐density plots illustrating the adsorption of H_2_O_2_ on NiCo_2_S_4_@UiO‐66 (B) without and (C) with US stimulation, in which red and green regions correspond to electron accumulation and depletion. d‐band center analysis of NiCo_2_S_4_@UiO‐66 under (D) non‐US and (E) US conditions. Free‐energy diagrams for (F) POD‐like activity and (G) the corresponding optimized stable structures.

To elucidate the underlying mechanisms of enzymatic and piezo‐catalytic enhancement in NiCo_2_S_4_@UiO‐66 system, density functional theory (DFT) calculations were performed. Given the computational complexity introduced by the large‐scale UiO‐66 clusters and the localization of peroxidase‐like active sites on NiCo_2_S_4_, the theoretical model was appropriately simplified. Specially, NiCo_2_S_4_ models with and without an applied electric field were adopted to represent the system under US and non‐US conditions, respectively. The optimized H_2_O_2_ molecule exhibited an O─O bond length of 1.4708 Å (Figure 3A). Differential charge density and Bader charge analyses were subsequently conducted to investigate the adsorption and activation behavior of H_2_O_2_ in the presence and absence of US stimulation. As shown in Figure 3B,C, strong adsorption of the reactant induced pronounced charge transfer between NiCo_2_S_4_ and H_2_O_2_ via an acceptor‐donor interaction mechanism. In the differential charge density maps, significant interfacial charge redistribution was observed, wherein empty Co‐3d orbitals accepted lonepair electrons from H_2_O_2_, while occupied Co‐3d orbitals backdonate electrons to the adsorbate. Consistently, Bader charge analysis revealed enhanced electron transfer under US. H_2_O_2_ obtained 0.02 e^−^ from NiCo_2_S_4_@UiO‐66 without US, whereas under US the charge transfer increased to 0.04 e^−^. This greater electron accumulation on the reactant was expected to facilitate subsequent catalytic reaction steps. Accordingly, the O─O bond length of adsorbed H_2_O_2_ elongated from 1.4768 Å (without US) to 1.4896 Å (under US), indicating more pronounced bond activation. These results confirmed that H_2_O_2_ could be effectively adsorbed and activated on the NiCo_2_S_4_ surface, and that US stimulation further promoted O─O bond elongation. Such synergistic structural effects contributed to the enhanced catalytic performance of the NiCo_2_S_4_@UiO‐66 system. Moreover, the electronic density of states (DOS) of NiCo_2_S_4_@UiO‐66 was analyzed both with and without US excitation (Figure 3D,E). In both cases, the DOS crossed the Fermi level (0 eV), indicating favorable electrical conductivity. Notably, the Co d‐band center was consistently higher than that of Ni, implying stronger adsorption capability and identifying Co as the primary active site for H_2_O_2_ catalysis. Upon US excitation, the Co d‐band center shifted downward from −1.32 to −1.44 eV. Given that ^*^OH desorption constituted the thermodynamic rate‐determining step for OH radical generation from H_2_O_2_, such a downward shift facilitated ^*^OH desorption and thereby improved reaction thermodynamics. Regarding the POD‐like catalytic pathway (Figure 3F,G), the adsorption of H_2_O_2_ on the NiCo_2_S_4_ surface yielded a Gibbs free energy change (ΔG) of −3.43 eV in the absence of US. Under US conditions, ΔG became slightly less negative (−3.12 eV), indicating that H_2_O_2_ dissociation remained thermodynamically favorable in both cases. However, the conversion of adsorbed ^*^OH to free OH radicals required additional energy input and represented the thermodynamic rate‐determining step. The energy barrier for OH radical generation was significantly reduced under US conditions compared to non‐US. These results demonstrated that NiCo_2_S_4_@UiO‐66 exhibited a substantially reduced thermodynamic barrier for the POD‐like reaction under ultrasonic stimulation, leading to more efficient catalytic kinetics as shown in the optimized stable structures along the enzymatic reaction pathway (Figure 3G).

### 2.3 Antimicrobial Resistance of NiCo_2_S_4_@UiO‐66

The growing threat of antimicrobial resistance, accelerated by antibiotic overuse, underscores the urgent need for effective therapeutic alternatives [35]. To this end, we selected multidrug‐resistant *Escherichia coli* (MDR *E. coli*) and methicillin‐resistant *Staphylococcus aureus* (*S. aureus)* (*MRSA*) as representative clinical models of resistant bacteria, and 0.5 W cm^−2^ (10 min) as the US exposure dosage referring to the U.S. Food and Drug Administration (FDA) safety guidelines [36, 37, 38]. In addition, we performed infrared thermal imaging of the NiCo_2_S_4_@UiO‐66 dispersion and the local skin tissue of mice to evaluate the potential thermal effect induced by our experimental US conditions. The temperature of the NiCo_2_S_4_@UiO‐66 dispersion increased by ∼13°C as the US duration was extended to 10 min (Figure S9A). In the mouse skin tissue experiment, the dorsal skin temperature was ∼36°C without US irradiation, but increased to ∼43°C under US irradiation (Figure S9B). These results indicated that 0.5 W cm^−2^ (10 min) had good tissue tolerance and safety, which were far below the threshold for irreversible thermal injury [39].

**FIGURE 4:**
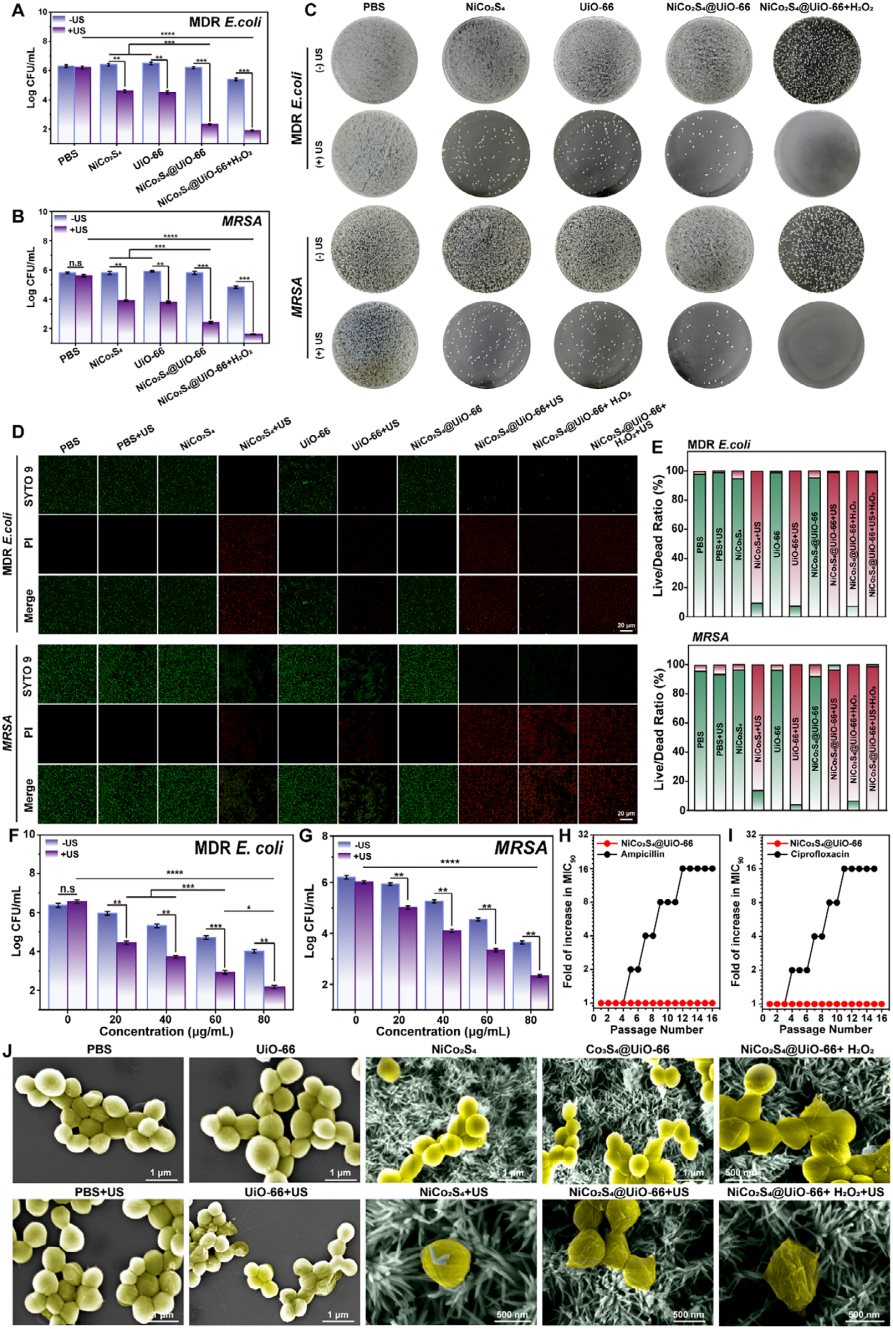
Survival (A) MDR *E. coli* cell counts and (B) *MRSA* cell counts (in Log CFU mL^−1^, where CFU means colony forming unit) after treatment with different groups in PBS with and without US exposure (at 0.5 W cm^−2^ for 10 min) (n = 3 independent replicates, mean ± SD). (C) Images of MDR *E. coli* and *MRSA* colonies on agar plates after different treatments. (D) Representative CLSM images for a Live/Dead bacterial viability assay of MDR *E. coli* and *MRSA* suspension under different treatments, scale bar: 20 µm. (E) Quantitative analysis of dead (red) and live (green) bacteria of *MRSA* and *E. coli* in different treatment groups. Survival (F) MDR *E. coli* cell counts and (G) *MRSA* cell counts (in Log CFU mL^−1^, where CFU means colony forming unit) after treatment with a NiCo_2_S_4_@UiO‐66 at differing concentrations in PBS with and without US exposure (at 0.5 W cm^−2^ for 10 min) (n = 3 independent replicates, mean ± SD). Folds of increase in MIC_90_ (the minimum concentration to inhibit the growth of 90% inoculated (H) *E. coli* and (I) *S. aureus* cells) of NiCo_2_S_4_@UiO‐66 through serial passages of growth inhibition assays. (J) Typical SEM images of *MRSA* after various treatments. Statistical analysis was carried out with a two‐way ANOVA with Tukey's multiple‐comparison test (^*^ *p* < 0.05, ^**^ *p* < 0.01, ^***^ *p* < 0.001, ^****^ *p* < 0.0001, n.s. represented no significance).

In the absence of US stimulation, NiCo_2_S_4_, UiO‐66, and NiCo_2_S_4_@UiO‐66 all exhibited negligible antibacterial activity against either MDR *E. coli* or *MRSA* (Figure 4A–C). Under US activation, however, their bactericidal efficacy enhanced markedly, in which NiCo_2_S_4_ and UiO‐66 alone achieved inactivation ratios of 97.5% and 88.9% against MDR *E. coli*, and 98.8% and 92.3% against *MRSA*, respectively. Notably, NiCo_2_S_4_@UiO‐66 under US reached over 99.9% antibacterial activity for both resistant strains (Figure 4A,B). Furthermore, leveraging the POD‐like activity of NiCo_2_S_4_, the antibacterial efficacy of NiCo_2_S_4_@UiO‐66 was further enhanced to 99.99% against both bacteria upon addition of 0.2 mm H_2_O_2_. Live/dead bacterial viability assays revealed that more than 99% of bacterial cells in the NiCo_2_S_4_@UiO‐66+US+H_2_O_2_ group displayed red PI fluorescence, indicating widespread cell death (Figure 4D,E). In contrast, other treatment groups remained predominantly stained with green SYTO‐9 fluorescence. Concentration‐dependent studies confirmed that NiCo_2_S_4_@UiO‐66+H_2_O_2_ achieved the highest bactericidal efficacy (>99.99%) against both strains at 80 µg mL^−1^ (Figure 4F,G). This excellent antibacterial performance against resistant bacteria stems from the synergistic interplay between SDT and the ROS antibacterial activity of nanozyme.

Next, in order to assess its potential for inducing resistance, we performed a 16‐cycle serial passage assay. Antibiotics such as amoxicillin and ciprofloxacin rapidly selected for resistance populations (Figure 4H,I). In contrast, the NiCo_2_S_4_@UiO‐66 system maintained strong antibacterial efficacy throughout the passages, indicating that this treatment did not readily induce bacterial resistance. Direct visualization of bacterial morphological damage further supported these findings (Figure 4J), *MRSA* cells in the PBS and PBS+US groups retained intact with typical spherical morphology. Similarly, negligible structural damage was observed after treatment with NiCo_2_S_4_ or UiO‐66 alone. Under combined stimulation, however, distinct patterns of cellular injury became evident. On the one hand, the NiCo_2_S_4_+US treatment induced distinct mechanical puncturing of the cell walls, whereas the UiO‐66+US treatment led to cellular collapse and shrinkage, consistent with ROS‐mediated damage. These structural changes were further exacerbated in the NiCo_2_S_4_@UiO‐66+US and NiCo_2_S_4_@UiO‐66+H_2_O_2_ groups, resulting in more extensive cellular collapse and shrinkage. The most severe destruction was observed in the NiCo_2_S_4_@UiO‐66+US+H_2_O_2_ group, which exhibited complete cell wall disintegration and loss of intracellular contents, highlighting the synergistic effect of mechanical disruption and oxidative stress.

These results demonstrated that NiCo_2_S_4_@UiO‐66 exerted its potent antimicrobial activity through a dual synergistic mechanism: Firstly, the spiny structure of NiCo_2_S_4_ mediated mechanical damage to the cell wall via US cavitation. Secondly, NiCo_2_S_4_ enhanced US‐induced electron–hole separation in UiO‐66, boosting its piezoelectric catalytic performance for ROS generation. Conversely, UiO‐66 generated a local electric field within NiCo_2_S_4_ that facilitated electron transfer and amplified its POD‐like activity.

### 2.4 Anti‐Biofilm Capability Mediated by NiCo_2_S_4_@UiO‐66

**FIGURE 5:**
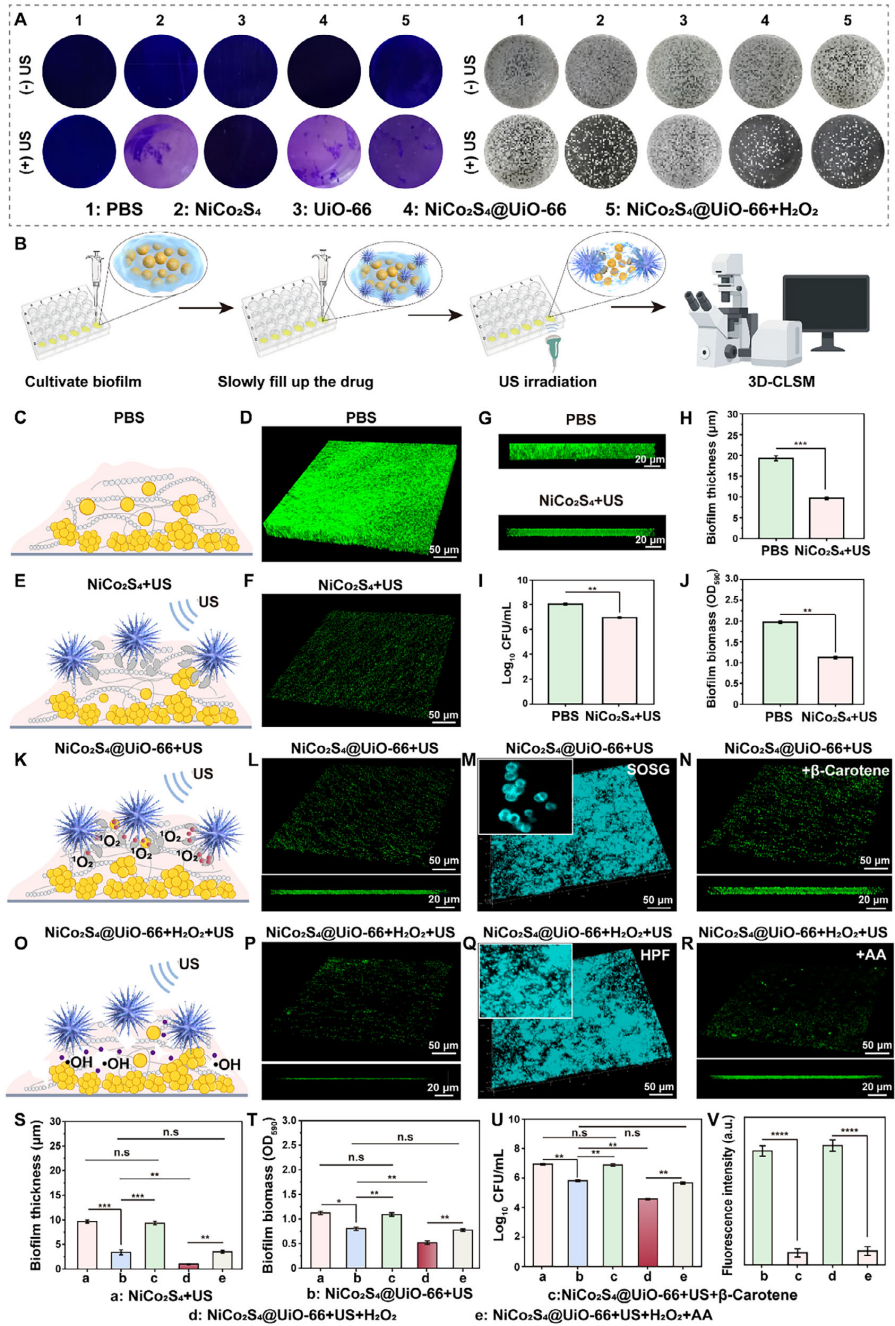
(A) Photographs of disrupted *MRSA* biofilms stained with crystal violet and images of *MRSA* colonies on agar plates after different treatments. (B) Schematic diagram of the ultrasound triggered by NiCo_2_S_4_@UiO‐66 to treat biofilm. (C) Schematic diagram of the *MRSA* biofilms and (E) biofilm treated with NiCo_2_S_4_+US. CLSM images showing the distribution of (D,G) PBS and (F,G) NiCo_2_S_4_@UiO‐66 in *MRSA* biofilms, with green fluorescence representing bacterial biofilm. (H) Biofilm thickness, (I) Cell counts (in Log CFU mL^−1^) and (J) Biofilm biomass of the US triggered by NiCo_2_S_4_@UiO‐66 to treat biofilm (n = 3 independent replicates, mean ± SD). (K) Schematic diagram of the *MRSA* biofilms treated with NiCo_2_S_4_@UiO‐66+US. (L) CLSM images showing the distribution of NiCo_2_S_4_@UiO‐66+US in *MRSA* biofilms, with green fluorescence representing bacterial biofilm. (M) CLSM images showing the SOSG by treated with NiCo_2_S_4_@UiO‐66+US in *MRSA* biofilms. (N) CLSM images showing the distribution of NiCo_2_S_4_@UiO‐66+US+β‐Carotene in *MRSA* biofilms. (O) Schematic diagram of the *MRSA* biofilms treated with NiCo_2_S_4_@UiO‐66+H_2_O_2_+US. (P) CLSM images showing the distribution of NiCo_2_S_4_@UiO‐66+H_2_O_2_+US in *MRSA* biofilms, with green fluorescence representing bacterial biofilm. (Q) CLSM images showing the HPF by treated with NiCo_2_S_4_@UiO‐66+H_2_O_2_+US in *MRSA* biofilms. (R) CLSM images showing the distribution of NiCo_2_S_4_@UiO‐66+H_2_O_2_+US+HPF in *MRSA* biofilms. (S) Biofilm thickness, (T) Cell counts (in Log CFU mL^−1^) and (U) Biofilm biomass after treatment with different groups (n = 3 independent replicates, mean ± SD). (V) Fluorescence intensity of SOSG and HPF after adding different quenchers. Statistical analysis was carried out with a one‐way ANOVA with Tukey's multiple‐comparison test. (^*^ *p* < 0.05, ^**^ *p* < 0.01, ^***^ *p* < 0.001, ^****^ *p* < 0.0001, n.s. represented no significance).

Bacterial biofilms pose a formidable therapeutic challenge, particularly in multidrug‐resistant infections, by impeding antibiotic penetration [40, 41]. Effective eradicating requires the dual action of disrupting the extracellular polymeric substance (EPS) matrix and eliminating the embedded bacteria [42, 43, 44]. We first evaluated the efficacy of NiCo_2_S_4_@UiO‐66 against preformed resistant bacterial biofilms in vitro. Crystal violet staining was performed to track the biofilm removal. As shown in Figure 5A, NiCo_2_S_4_@UiO‐66 exhibited superior biofilm‐disrupting activity under US stimulation, comparable to NiCo_2_S_4_ alone. Notably, with the addition of H_2_O_2_ under US stimulation, NiCo_2_S_4_@UiO‐66 nearly completely ablated the biofilm, reducing viable bacteria counts by over 99%. Further details of US‐mediated anti‐biofilm activity were examined using 3D confocal fluorescence imaging (Figure 5B). Confocal images confirmed that the formation of stable, dense *MRSA* biofilms after 48 h (Figure 5C,D), which remained unaffected by US alone, NiCo_2_S_4_, or NiCo_2_S_4_@UiO‐66 alone (Figure S10). Upon US activation, NiCo_2_S_4_ was transformed into a dynamic platform that mechanically penetrated and disrupted the biofilm (Figure 5E,F), resulting in a remarkable reduction in biofilm thickness from 19.6 to 9.9 µm (Figure 5G,H), along with decreased viable bacterial counts by 88.6%, and biofilm biomass by 40% (Figure 5I,J). In contrast, UiO‐66 failed to apparently alter biofilm structure even under US due to its limited penetration ability (Figure S11). Such ability to destroy biofilm was further enhanced by the NiCo_2_S_4_@UiO‐66 composite (Figure 5K,L).

Severe structural disintegration was observed, with biofilm thickness dramatically reduced to 3.8 µm (Figure 5S), accompanied by near‐total elimination of viable *MRSA* and biofilm mass (Figure 5T‐U). To assess the anti‐biofilm activity and distribution of ^1^O_2_ and ·OH in the biofilm microenvironment, we employed the specific probes SOSG and HPF to visualize their localization without cross‐talk (Figure S12). The presence of ^1^O_2_ throughout the biofilm was directly visualized via SOSG fluorescence, confirming the successful sonodynamic therapy within the pretreated biofilm (Figure 5M). When *MRSA* biofilm was incubated with the β‐carotene as ^1^O_2_ quencher, the SOSG fluorescence signal induced by NiCo_2_S_4_@UiO‐66 under US stimulation was nearly abolished. Correspondingly, the anti‐biofilm efficacy was weakened, resulting in higher residual film thickness (9.2 µm), biomass (1.12 of OD_590_), and bacterial counts (6.92 in Log CFU mL^−1^) compared to the original NiCo_2_S_4_@UiO‐66+US group (Figure 5N,S‐U). These results demonstrated that ^1^O_2_ generated by NiCo_2_S_4_@UiO‐66 via SDT action not only enhanced the disruption ability of biofilms, but also efficiently inactivated *MRSA* embedded in biofilm. Upon addition of H_2_O_2_, NiCo_2_S_4_@UiO‐66 exhibited the strongest biofilm ablation under US stimulation, further reducing the film thickness to 1.1 µm (Figure 5O–S). Similarly, taking hydroxyphenyl fluorescein (HPF) as a ·OH fluorescent indicator, widespread ·OH generation catalyzed by NiCo_2_S_4_@UiO‐66 was detected at the periphery of the biofilm upon H_2_O_2_ addition, accompanied by evident degradation of EPS matrix (Figure 5Q). When ·OH was quenched with ascorbic acid (AA), the antibacterial efficacy declined to 92.6% with increased film thickness (3.1 µm), and biomass (0.76 of OD_590_) observed (Figure 5V‐U). This confirmed that ·OH generated by NiCo_2_S_4_@UiO‐66 played a key role in extensive EPS breakdown and enhanced biofilm ablation. These results verified that microenvironment‐dependent functional ROS generation established an effective platform for achieving high antibacterial efficacy.

### 2.5 In Vivo Antibacterial Performance of NiCo_2_S_4_@UiO‐66

Biofilm‐associated infections are difficult to eradicate due to their dynamic development, with distinct stages exhibiting different structures and therapeutic sensitivities. Accordingly, antibiofilm strategies have shifted from overall bactericidal approaches to stage‐oriented interventions, where targeting stage‐specific barriers improves the eradication of mature biofilms [45]. Activatable ROS‐based antibacterial strategies have recently gained attention, as they allow on‐demand ROS generation in response to infection microenvironments or external stimuli, improving efficacy while minimizing nonspecific damage. For example, Xie et al. developed a bacteria‐targeting microneedle photosensitizer system that achieved efficient photodynamic eradication of multidrug‐resistant biofilms [46]. Our developed NiCo_2_S_4_@UiO‐66 piezoelectric nanozyme integrates stage‐oriented therapy with activatable ROS regulation. Combined with mechanical penetration, matrix degradation, and deep bacterial killing, it enables more effective disruption of mature biofilms.

The biosafety of NiCo_2_S_4_@UiO‐66 was first evaluated prior to in vivo studies. Hela cells maintained over 90% viability at a concentration of 200 µg mL^−1^ and live/dead staining confirmed intact cellular morphology and proliferation (Figure S13A–C). To further evaluate wound‐related skin cell biocompatibility, we assessed the effect of NiCo_2_S_4_@UiO‐66 on the viability of L929 cells, a mouse fibroblast cell line derived from subcutaneous connective tissue. As shown in Figure S13D, cell viability remained ∼90% after 24 h incubation even at 200 µg mL^−1^. Upon US irradiation, cell viability remained above 95% up to 125 µg mL^−1^ and declined to 88% at 150 µg mL^−1^ (Figure S13E), indicating negligible cytotoxicity below 150 µg mL^−1^ with or without US. This conclusion was further confirmed by a lactate dehydrogenase (LDH) release assay (Figure S13F,G). Prolonged exposure (80 µg mL^−1^, 24–72 h) caused no significant viability loss under either condition, indicating stable cytocompatibility (Figure S13H) under extended incubation conditions. In vivo, NiCo_2_S_4_@UiO‐66 showed no obvious biotoxicity in a full‐thickness skin‐defect model as evidenced by stable body weight, unchanged serum inflammatory markers, normal blood count parameters, and unaltered liver and renal function indicators, as well as no obvious pathological abnormalities in the major organs (Figure S14).

**FIGURE 6:**
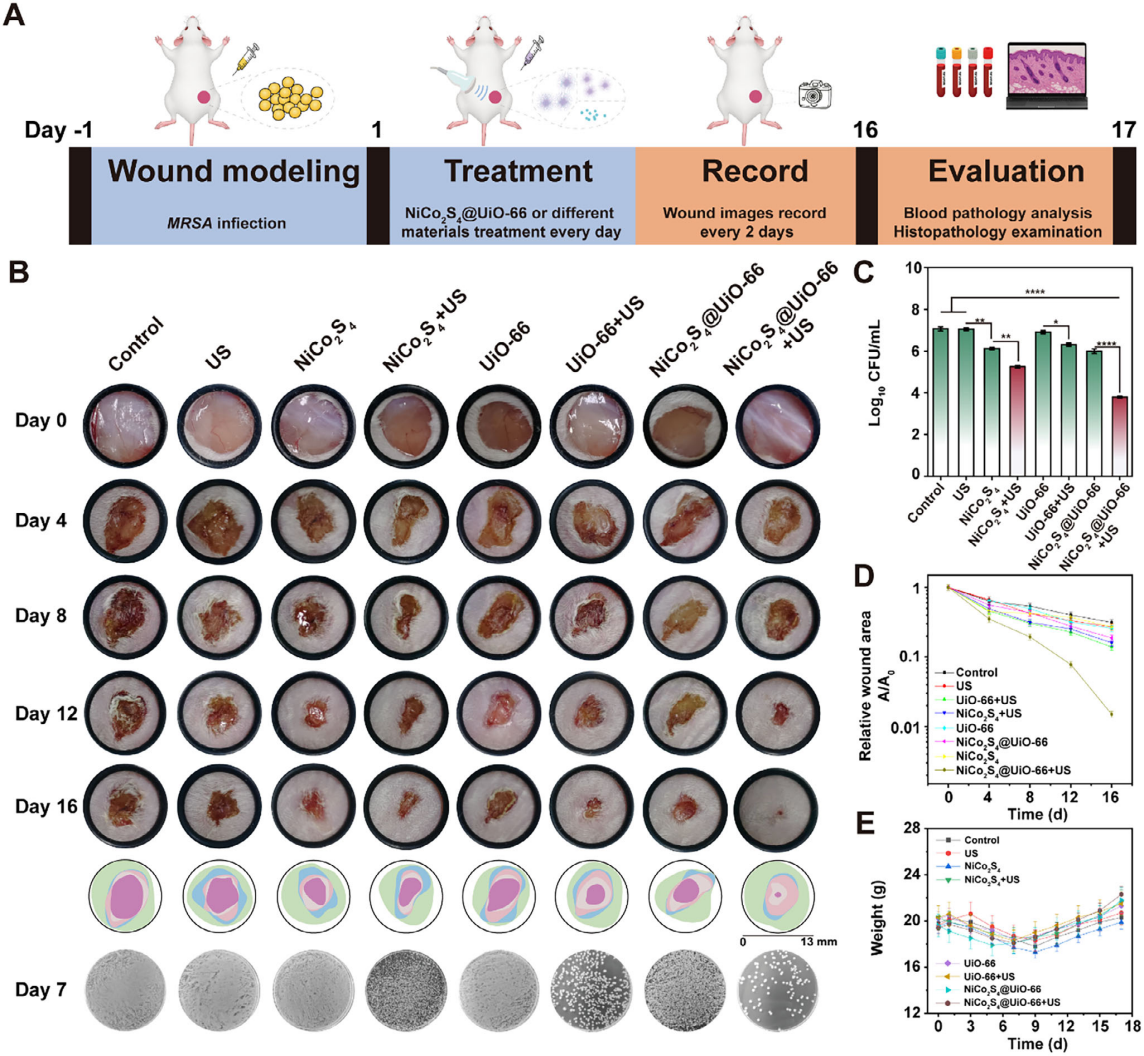
(A) Schematic illustration of the treatment schedule for the *MRSA* infected wounds. (B) Digital photographs of wound sizes on different days and *MRSA* colonies that were harvested from different groups on day 7. (C) The counted bacterial colony numbers in (B) (n = 3 biologically independent replicates, mean ± SD). (D) Quantitative analysis of relative wound area during the various treatment periods (n = 5 biologically independent mice in one trial, mean ± SD). (E) Relative body weight changes during the various treatment periods (n = 5 biologically independent mice in one trial, mean ± SD). Statistical analysis was carried out with a one‐way ANOVA with Tukey's multiple‐comparison test. (^*^ *p* < 0.05, ^**^ *p* < 0.01, ^***^ *p* < 0.001, ^****^ *p* < 0.0001).

Next, the therapeutic efficacy against drug‐resistant biofilm infections was evaluated in vivo. A mouse model of *MRSA* biofilm infection was first established using full‐thickness skin defects (Figure 6A). Wounds in BALB/c mice were inoculated with 10^8^ CFU mL^−1^ *MRSA* and allowed to form biofilms over two days. Subsequently, the wounds were treated for sixteen days with PBS (Control), US alone, NiCo_2_S_4_, UiO‐66, NiCo_2_S_4_@UiO‐66, and their corresponding states with US stimulation were administered. Wound progression was monitored every two days, and mice were sacrificed on day 17th for analysis. The US and UiO‐66 groups exhibited similar healing rates, with substantial bacterial presence still detectable at day 7 (Figure 6B,C) and large residual wound areas at day 16 (Figure 6D). In contrast, treatments with NiCo_2_S_4_, NiCo_2_S_4_+US, UiO‐66+US, and NiCo_2_S_4_@UiO‐66 partially suppressed bacteria and accelerated healing, though significant bacterial loads remained at day 7 and would closure reached only over 50% by day 16. Notably, the NiCo_2_S_4_@UiO‐66+US treatment led to a 99.9% reduction in bacterial counts by day 7 and achieved near‐complete wound closure by day 16 (Figure 6B–D). Additionally, no significant body weight changes were observed across groups, indicating negligible systemic toxicity (Figure 6E). These findings demonstrate that US‐activated NiCo_2_S_4_@UiO‐66 effectively eradicates *MRSA* biofilms and markedly accelerates wound healing in vivo.

**FIGURE 7:**
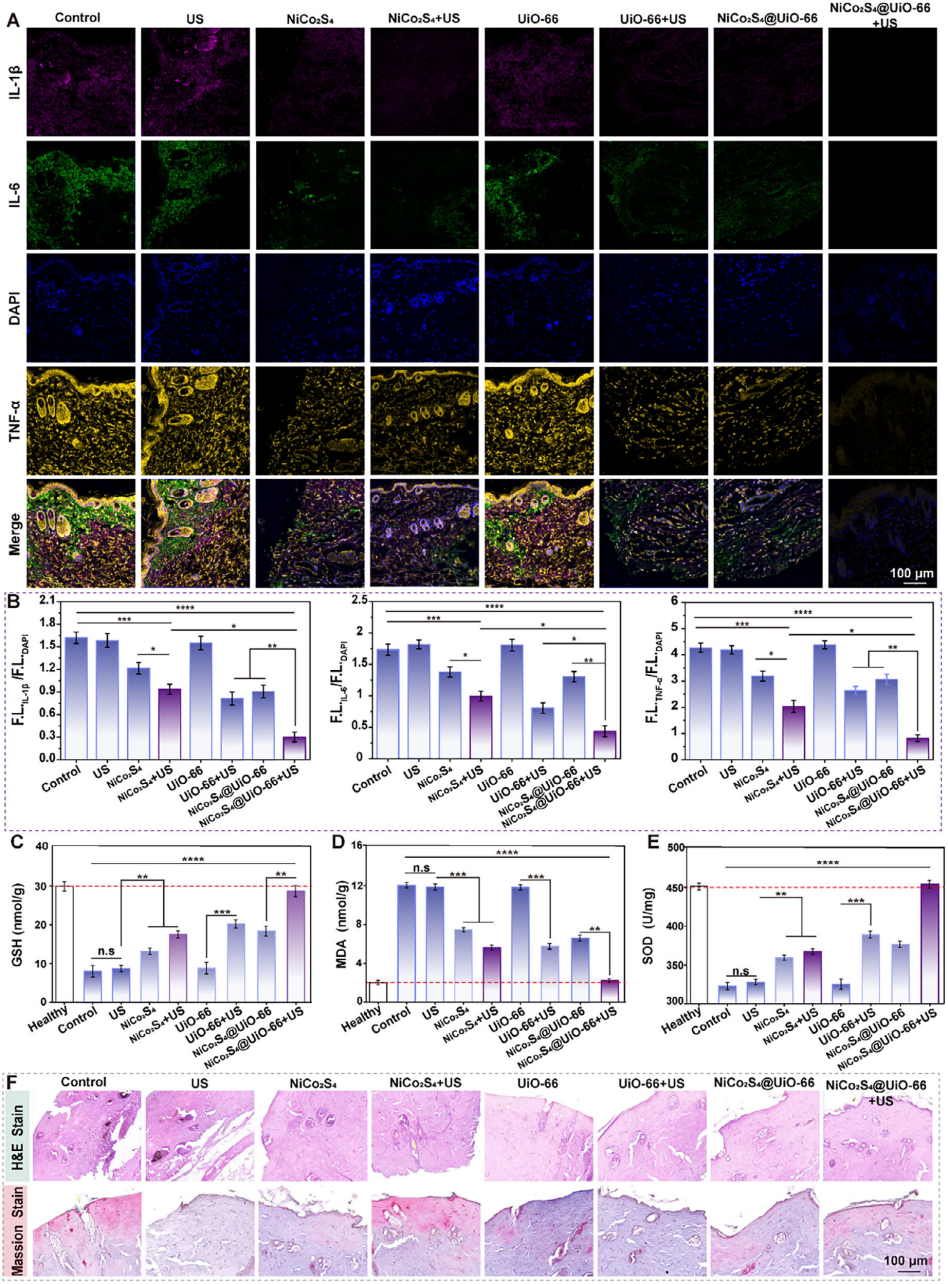
(A) Immunofluorescent staining of peripheral tissues from each group. Red fluorescence indicates IL‐1β, green fluorescence indicates IL‐6, yellow fluorescence indicates TNF‐α and blue fluorescence represents nuclei (Scale bar, 100 µm). (B) Corresponding quantitative analyses of the mean fluorescence intensity of IL‐1β, IL‐6 and TNF‐α (n = 5 biologically independent mice in one trial, mean ± SD). Serum levels of (C) GSH, (D) MDA and (E) SOD in *MRSA* infection‐bearing mouse models after differing treatments (n = 5 biologically independent mice in one trial, mean ± SD). (F) H&E and Masson staining images of the epidermal histological sections in different groups after treatment. Statistical analysis was carried out with a one‐way ANOVA with Tukey's multiple‐comparison test. (^*^ *p* < 0.05, ^**^ *p* < 0.01, ^***^ *p* < 0.001, ^****^ *p* < 0.0001, n.s. represented no significance).

To investigate the physiological mechanisms underlying the therapeutic effects, we analyzed key pro‐inflammatory cytokines (IL‐6, IL‐1β, and TNF‐α), which regulate inflammatory responses and immune cell activity [38, 47]. These substances dysregulation can hinder wound repair. Assessment of serum cytokine expression revealed that treatments with NiCo_2_S_4_, NiCo_2_S_4_+US, UiO‐66+US, and NiCo_2_S_4_@UiO‐66 resulted in only partial reductions. In contrast, the NiCo_2_S_4_@UiO‐66+US treatment reduced cytokine levels by more than twofold compared to the control group (*p* < 0.0001), restoring them to baseline levels comparable to those in healthy mice (Figure S15). This finding indicated a pronounced systemic anti‐inflammatory effect. We further tracked the local wound microenvironment using immunofluorescence staining. Following NiCo_2_S_4_@UiO‐66+US treatment, the expression of IL‐1β, IL‐6, and TNF‐α at the wound site decreased dramatically by 5.1‐, 4.2‐, and 4.4‐fold, respectively (*P* < 0.0001) (Figure 7A,B). These data suggested that the therapy effectively mitigated both systemic and local inflammation, thereby normalizing the wound healing environment.

Next, we evaluated oxidative stress levels at the wound site, an important factor that impairs healing during infection. We quantified biomarkers representing distinct facets of oxidative injury and defense: Glutathione (GSH) is a key intracellular antioxidant that preserves redox homeostasis by scavenging ROS and regulating oxidative stress; malondialdehyde (MDA), a terminal product of lipid peroxidation; and superoxide dismutase (SOD), a primary antioxidant enzyme whose depletion exacerbates oxidative injury [48, 49, 50, 51, 52]. While treatments with NiCo_2_S_4_, NiCo_2_S_4_+US, UiO‐66+US, and NiCo_2_S_4_@UiO‐66 resulted in partial amelioration, the NiCo_2_S_4_@ UiO‐66+US regimen yielded a more than two‐fold reduction in MDA levels, while restoring GSH levels and SOD activity to near‐normal levels (*p* < 0.0001) (Figure 7C–E). These data indicated effective reversal of the infection‐driven oxidative imbalance. Histological analysis using hematoxylin and eosin (H&E) and Masson's trichrome staining further confirmed enhanced wound healing. H&E staining showed gradual restoration of epidermal integrity, increased epidermal thickness, basal cell regeneration, and stratum corneum formation, and evidence of re‐epithelialization in all treatment groups. Masson's staining revealed markedly elevated collagen deposition in the NiCo_2_S_4_@UiO‐66+US group on day 11 (Figure 7F). These results demonstrated that NiCo_2_S_4_@UiO‐66+US treatment effectively remodeled the wound microenvironment by concurrently attenuating inflammation, reducing oxidative stress, and promoting the structural regeneration of skin tissue.

**FIGURE 8:**
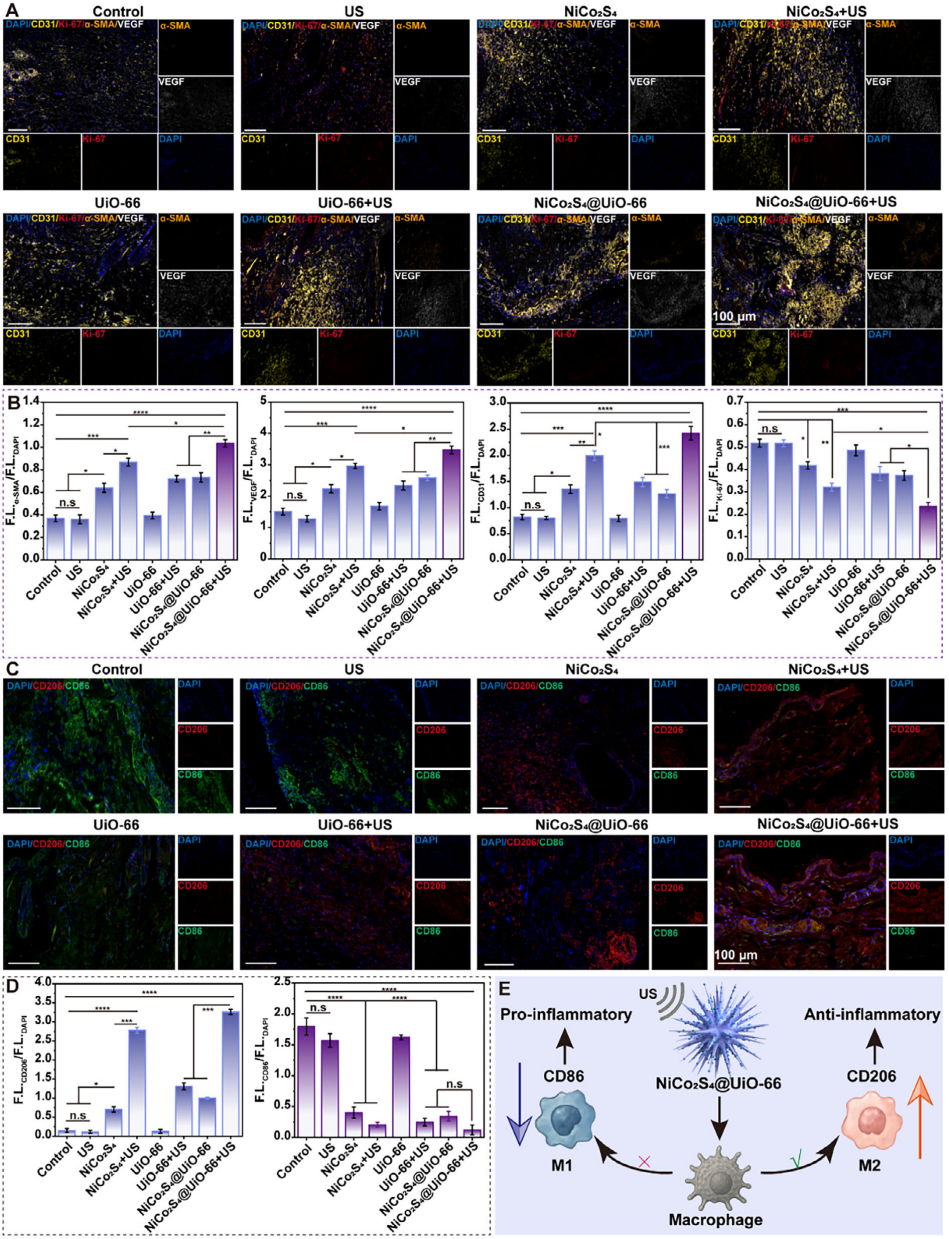
(A) Immunofluorescent staining of peripheral tissues from each group. Red fluorescence indicates Ki‐67, white fluorescence indicates VEGF, yellow fluorescence indicates CD31, orange fluorescence indicates α‐SMA, and blue fluorescence represents nuclei (Scale bar, 100 µm). (B) Corresponding quantitative analyses of the mean fluorescence intensity of Ki‐67, CD31, VEGF, and α‐SMA (n = 5 biologically independent mice in one trial, mean ± SD). (C) Immunofluorescent staining of peripheral tissues from each group. Red fluorescence indicates CD206, green fluorescence indicates CD86, and blue fluorescence represents nuclei (Scale bar, 100 µm). (D) Corresponding quantitative analyses of the mean fluorescence intensity of CD86 and CD206 (n = 5 biologically independent mice in one trial, mean ± SD). (E) Schematic diagram of NiCo_2_S_4_@UiO‐66 regulating the immune microenvironment at the wound site. Statistical analysis was carried out with a one‐way ANOVA with Tukey's multiple‐comparison test. (^*^ *p* < 0.05, ^**^ *p* < 0.01, ^***^ *p* < 0.001, ^****^ *p* < 0.0001, n.s. represented no significance).

To elucidate the therapeutic mechanism of NiCo_2_S_4_@UiO‐66+US in infected wounds and its molecular basis for promoting healing, we employed immunofluorescence (IF) staining to analyze key protein markers (Figure 8). Cluster of differentiation 31 (CD31) [53], vascular endothelial growth factor (VEGF) [54], α‐smooth muscle actin (α‐SMA) [55], and Ki‐67 [56] are essential biomarkers in skin wound healing. CD31, an endothelial marker, reflects angiogenesis and new blood vessel formation at the wound site, which is critical for tissue regeneration [57]. VEGF, a pro‐angiogenic factor, promotes endothelial cell proliferation and vascularization, thereby enhancing nutrient and oxygen delivery to healing tissue [58]. The α‐SMA, expressed in myofibroblasts, indicates wound contraction and tissue remodeling [59]. Ki‐67, a proliferation marker, assesses the activity of keratinocytes and fibroblasts, both essential for reepithelialization and tissue regeneration [60]. These biomarkers were chosen to provide insights into angiogenesis, fibroplasia, and cell proliferation during wound healing. No significant changes in CD31, VEGF, α‐SMA, or Ki‐67 expression were observed in the US and UiO‐66 groups. In contrast, treatments with NiCo_2_S_4_, NiCo_2_S_4_+US, UiO‐66+US, and NiCo_2_S_4_@UiO‐66 induced moderate increases in CD31, VEGF, and α‐SMA levels, accompanied by a slight decrease in Ki‐67 (Figure 8A,B). Moreover, NiCo_2_S_4_@UiO‐66+US treatment resulted in 2.5, 2.3, and 3.2‐fold upregulation in α‐SMA, VEGF, and CD31 expression, respectively (*p* < 0.0001), and a 2.1‐fold downregulation in Ki‐67 (*p* < 0.001) (Figure 8A,B). These results suggest that the treatment enhances angiogenesis, thereby improving nutrient and energy supply to the wound site and accelerating skin barrier restoration. Simultaneously, the reduction in Ki‐67 indicates suppression of excessive cell proliferation, which may contribute to minimized scar formation during healing.

Macrophage phenotype switching plays dual‐role in inflammation that M1 macrophages mediate acute‐phase anti‐infection responses, while M2 macrophages resolve inflammation and promote tissue repair. Staining for macrophage phenotypes revealed that NiCo_2_S_4_@UiO‐66+US treatment significantly increased CD206 (M2, red fluorescence) and reduced CD86 (M1, green fluorescence) expression, while the control group showed the opposite pattern. This indicates that NiCo_2_S_4_@UiO‐66+US promotes M1‐to‐M2 macrophage polarization (Figure 8C,D). NiCo_2_S_4_@UiO‐66+US treatment induces immune reprogramming, facilitating the M1‐to‐M2 macrophage switch and precisely regulating the transition from acute inflammation to tissue repair (Figure 8E). This mechanism underlies the observed improvement in the prognosis of infected wounds treated with NiCo_2_S_4_@UiO‐66+US. Assessing host toxicity remains a primary concern in therapeutic development. To evaluate the biocompatibility of the NiCo_2_S_4_@UiO‐66‐based mechano‐catalyzed sonodynamic nanozyme therapy, we analyzed liver and renal function in a murine model of *MRSA*‐infected wounds 48 h post‐treatment (Figure S16). Serum levels of alanine aminotransferase (ALT), aspartate aminotransferase (AST), and albumin (ALB) exhibited transient elevations following NiCo_2_S_4_@UiO‐66+US treatment but returned to baseline, indicating no obvious liver damage (Figure S16A–C). Similar results were observed for blood urea nitrogen (BUN), creatinine (CREA), and uric acid (UA) levels (Figure S16D–F), suggesting no renal dysfunction. Furthermore, histological examination of major organs (heart, liver, spleen, lungs, and kidneys) via H&E staining revealed no structural abnormalities or pathological lesions (Figure S17). These results demonstrated that NiCo_2_S_4_@UiO‐66 mechanochemically mediated sonodynamic nanozyme therapy is highly biocompatible, providing a safe and effective novel approach for treating drug‐resistant biofilm infections.

## 3 Conclusions

In summary, we present an innovative strategy to address biofilm‐associated infections caused by antibiotic‐resistant bacteria, tackling the challenges of therapeutic resistance and resistance propagation. By combining surface‐spiked nanozymes with piezoelectric MOF materials (NiCo_2_S_4_@UiO‐66), we have developed a system that integrates US‐triggered mechanical force enhancement with a bidirectional synergistic mechanism of SDT and POD enzyme activity. Upon US activation, the enhanced mechanical forces enable NiCo_2_S_4_@UiO‐66 to penetrate and infiltrate biofilms, inducing mechanical cell death in a substantial number of bacteria. Concurrently, NiCo_2_S_4_@UiO‐66 generates localized high concentrations of ^1^O_2_ around the bacteria within the biofilm, exploiting the long‐lived and diffusible nature of ^1^O_2_ to induce secondary, highly efficient bacterial death. Furthermore, in the presence of high concentrations of hydrogen peroxide at the wound site, NiCo_2_S_4_@UiO‐66 exhibits enhanced POD enzyme activity, generating significant quantities of ·OH both within and outside the biofilm. This leads to widespread degradation of the EPS matrix, resulting in effective biofilm eradication. In a mouse model of *MRSA*‐infected wounds, NiCo_2_S_4_@UiO‐66 not only demonstrated potent antimicrobial properties but also accelerated wound healing by modulating the inflammatory environment, reducing oxidative stress and promoting neovascularization. This approach provides a promising antibiotic‐free strategy, introducing a surface‐spiked piezoelectric nanozyme composite that leverages US‐triggered mechanical force and a bidirectional synergistic ROS release mechanism to disrupt biofilms and directly eliminate bacteria. This advanced antimicrobial system overcomes the challenges posed by biofilms, offering enhanced treatment for antibiotic‐resistant biofilm infections and presenting potential applications for other chronic wound infections.

## 4 Experimental Section

### 4.1 Synthesis of NiCo_2_S_4_@UiO‐66

The Ni‐Co (OH)_2_@NF precursor was synthesized following the Supporting Information's method. Subsequently, for the vulcanization stage, UiO‐66 and Na_2_S·9H_2_O were simultaneously introduced into a Teflon‐lined stainless‐steel autoclave. The sealed autoclave was then maintained at 90°C for 12 h. Upon completion, the NiCo_2_S_4_@UiO‐66@NF composite was obtained.

### 4.2 Bacterial Plate‐Killing Assays with NiCo_2_S_4_@UiO‐66

Bactericidal activity of NiCo_2_S_4_@UiO‐66 was evaluated using plate bacterial killing assays. Representative Gram‐positive bacterial strains *MRSA* (ATCC 43300) and Gram‐negative bacterial strains MDR *E. coli* (ATCC 35218) were tested, as an antibiotic‐resistant bacterial strain. For each strain, 3–5 individual colonies were inoculated into fresh TSB and incubated at 37°C for 16–18 h. A 40 µL aliquot of each culture was diluted with fresh TSB by 100‐fold and regrown at 37°C to mid‐log phase (OD_600_ = 0.5–0.7). Bacterial cells were harvested, washed three times with PBS via centrifugation (10 000 g for 5 min at 4°C), and resuspended in PBS to ∼1.5 × 10^6^ CFU mL^−1^. The bacterial suspension (50 µL) was inoculated into zero‐dilution wells of a 96‐well microplate. Serial twofold dilutions of NiCo_2_S_4_@UiO‐66 dispersion were prepared in PBS buffer. Each dilution (100 µL) was added into the corresponding wells, followed by the inoculation of 50 µL of bacterial suspension into each well to achieve a final concentration of 5 × 10^5^ CFU mL^−1^ (150 µL). The microplate was treated with different groups. tenfold serial dilutions were subsequently made with PBS buffer, followed by plating the dilutions (20 µL) onto TSB agar plates for overnight incubation at 37°C to form visible colonies. Inoculum size was indicated by control samples containing bacteria treated similarly but without NiCo_2_S_4_@UiO‐66. The minimum bactericidal concentration (MBC) value was defined as the minimum concentrations of NiCo_2_S_4_@UiO‐66 or antibiotics required to inhibit 99.9% bacterial growth.

### 4.3 SEM Characterizations on the Bacterial Morphology after NiCo_2_S_4_@UiO‐66 Treatment

Briefly, 3–5 individual colonies were inoculated into fresh TSB and incubated at 37°C for 16–18 h to reach the stationary phase. A 40 µL culture was diluted 100‐fold in fresh TSB and regrown at 37°C to mid‐log phase (OD_600_ = 0.5–0.7). Bacterial cells were harvested, washed three times with PBS by centrifugation (10 000 g for 5 min at 4°C), and resuspended in PBS to ∼1.5 × 10^8^ CFU mL^−1^. NiCo_2_S_4_@UiO‐66 dispersion was added into bacteria suspension, achieving a final bacterial concentration of ∼10^8^ CFU mL^−1^ and a final NiCo_2_S_4_@UiO‐66 mass concentration (80 µg mL^−1^). The mixture was treated with different groups, followed by centrifugation at 10 000 g for 5 min to remove the supernatant. The pellet was fixed with 4% formaldehyde at 4°C for 1 h, and then dehydrated in graded ethanol solutions (25%, 50%, 75%, 90%, and 100%) via centrifuge (10 000 g for 5 min). The final pellet was resuspended in 100 µL ethanol (100%), and 10 µL of the resulting dispersion was dropped onto a copper tape and dried overnight. The sample was then sputtered with gold and imaged using a SEM.

### 4.4 Bacterial Dead/Live Viability Assays with NiCo_2_S_4_@UiO‐66

Bacterial viability was assessed using a live/dead bacterial viability kit, and staining was examined under fluorescence microscopy (Leica). SYTO‐9 and propidium iodide (PI) were used to label live and dead cells, respectively. MDR *E. coli* (ATCC 35218) served as representative Gram‐negative bacteria strains, while *MRSA* (ATCC 43300) was used as representative Gram‐positive bacteria. For each bacterial strain, 3–5 individual colonies were inoculated into fresh TSB and incubated at 37°C for 16–18 h to reach stationary phase. A 40 µL culture was diluted 100‐fold in fresh TSB and regrown at 37°C to mid‐log phase (OD_600_ = 0.5–0.7). Then, bacterial cells were harvested, washed once with PBS via centrifugation (10 000 g for 5 min at 4°C), and resuspended in sterile PBS to ∼1.5 × 10^8^ CFU mL^−1^. The NiCo_2_S_4_@UiO‐66 dispersion was added into the as‐adjusted bacteria suspension, achieving a final bacterial inoculum concentration of ∼1 × 10^8^ CFU mL^−1^ and a final mass concentration of 80 µg mL^−1^. The mixing suspension was then treated with different groups. Following treatment, 100 µL of the treated bacterial suspension was stained with 5 µL of SYTO‐9 and 5 µL of PI for 15 min in the dark, then centrifuged at 10 000 g for 5 min to remove the supernatant. The bacterial pellets were then washed with 100 µL of PBS. The resultant bacterial suspension was transferred to a coverslip, air‐dried, and immersed in PBS, before being imaged under fluorescence microscopy. Each sample was imaged at 15 different areas, and quantitative analysis on the microscopy images was performed.

### 4.5 In Vitro Antibiofilm Assays

Biofilms were established by adding 100 µL of cultivated *MRSA* suspensions (1 × 10^8^ CFU mL^−1^) to 900 µL of TSB medium in a 24‐well plate, followed by incubation at 37°C for 48 h to allow mature biofilm formation. The preformed biofilms were subsequently treated with different groups and incubated at 37°C for an additional 48 h. After treatment, the supernatant was carefully removed, and the remaining biofilms were gently washed three times with PBS (pH 7.4). Biofilm biomass was then assessed by crystal violet staining and quantitatively analyzed using enzymatic detection. For viability analysis, biofilms from the four groups were stained with SYTO‐9 for 30 min at room temperature and imaged by confocal laser scanning microscopy (CLSM) to obtain 3D fluorescence images.

### 4.6 Statistical Analysis

All the statistical analyses were performed using GraphPad Prism 8 (GraphPad Software, Inc.). Statistical comparisons were carried out by performing a two‐sided Student's t‐test, one‐way and two‐way ANOVA with Tukey's multiple‐comparison test, ^*^ *p* < 0.05, ^**^ *p* < 0.01, and ^***^ *p* < 0.001, respectively.

## Author Contributions

**Jin Yang**: writing – review and editing, data curation, supervision, and software. **Limin Zhang**: methodology, writing – review and editing, writing – original draft, investigation, funding acquisition, project administration, resources. **Xinjian Guo**: software, conceptualization, investigation, writing – original draft, data curation, formal analysis, validation. **Tao Liu**: visualization, writing – review and editing, writing – original draft, conceptualization, methodology, data curation, supervision, resources, funding acquisition. **Mengjie An**: visualization, data curation. **Bingjie Lin**: investigation, formal analysis, validation.

## Funding

Natural Science Foundation of China (22022402, 21974051 for L. M. Zhang), East China Normal University‐Shanghai Putuo District Central Hospital Collaborative Research Center for Translational Medicine, East China Normal University (ECNU‐SPDH CCTM‐202510), and the China Postdoctoral Science Foundation under Grant Number 2025M771003 and the Postdoctoral Fellowship Program of CPSF under Grant Number GZC20240477.

## Conflicts of Interest

The authors declare no conflict of interest.

## Supporting information




**Supporting File**: advs76086‐sup‐0001‐SuppMat.docx.

## Data Availability

The data that support the findings of this study are available from the corresponding author upon reasonable request.
