# Supplementary material for: Inside‐Outside ROS Therapeutic Strategy Based on Piezoelectric Nano‐Urchin for Drug‐Resistant Bacteria Biofilm Infections
Source: Adv Sci (Weinh). 2026 Jun 12;13(50):e76086. doi: 10.1002/advs.76086 (PMC13336586; doi:10.1002/advs.76086)
Supplement: Supplementary file 1 [file ADVS-13-e76086-s001.docx]

Supporting Information

**Inside-outside ROS Therapeutic Strategy Based on Piezoelectric Nano-urchin for Drug-Resistant Bacteria Biofilm Infections**

Xinjian Guo^1^, Jin Yang^2,^*, Mengjie An^1^, Bingjie Lin^1^, Tao Liu^1,^*, and Limin Zhang ^1,^*

^1^ Shanghai Key Laboratory of Green Chemistry and Chemical Processes, School of Chemistry and Molecular Engineering, East China Normal University, Dongchuan Road 500, Shanghai 200241, P.R. China

^2^ Institute of Cardiovascular Translational Medicine, Putuo Hospital, Shanghai University of Traditional Chinese Medicine, Shanghai, 200062, China

**1.Materials and Methods**

*Materials***:** Cobalt (II) nitrate hexahydrate (Co (NO_3_)_2_·6H_2_O, 99%), urea (CO(NH_2_)_2_, 99.5%), urea (CO(NH_2_)_2_, 99.5%) and 3,3’,5,5’-tetramethylbenzidine (TMB) were acquired from Sigma-Aldrich (USA). Sodium sulfide nonahydrate (Na_2_S·9H_2_O) was acquired from Macklin Biochemical Co., Ltd (Shanghai, China). 2’,7’-Dichlorodihydrofluorescein diacetate (DCFH-DA) and pure phthalic acid (PTA) were purchased from Yuanye BioTechnology Co., Ltd (Shanghai, China). Hydroxyphenyl fluorescein was purchased from MedChemExpress (USA). Columbia agar broth powder and tryptic soy broth (TSB) were purchased from Qingdao Hope BioTechnology Co., Ltd (Qingdao, China). Fetal bovine serum (FBS) was purchased from ExCell Biology. Inc (Shanghai, China). For alanine aminotransferase (ALT), aspartate aminotransferase (AST), albumin (ALB), creatinine (CREA), uric acid (UA)，blood urea nitrogen (BUN) enzyme-linked immunosorbent assay (ELISA) kits, singlet oxygen assay kit with SOSG and cell counting kit-8 (CCK-8) were purchased from Beyotime Biotechnology Co., Ltd (Shanghai, China). All other reagents were purchased from Sinopharm Chemical Reagent Company (Shanghai, China). All reagents were used as received without further purification unless otherwise noted. The nickel foam (NF, thickness: 1.5 mm; aperture: 0.2-0.25 mm; porosity: 95-98%) used in this work has a thickness of 1.0 mm and obtained from Youveim (China). The NF was cleaned three times with ethanol and deionized (DI) water then dried.

*Synthesis of NiCo_2_S_4_*: NiCo_2_S_4_ was synthesized following a procedure previously reported with some modifications.^[1]^ Briefly, 5.0 mmol of Co (NO_3_)_2_·6H_2_O, 5.0 mmol of Ni (NO_3_)_2_·6H_2_O, 30.0 mmol of CO (NH_2_)_2_, and 12.5 mmol of NH_4_F were dissolved in the DI water (10 ml) in a Teflon-lined stainless autoclave. Next, a cleaned and dried NF (10 × 50 mm^2^) was immersed in the solution. The hydrothermal reaction was maintained at 120 °C for 6 h. The obtained Ni-Co (OH)_2_@NF precursor was washed three times with DI water and ethanol. Then, 250 mg of Na_2_S·9H_2_O was dissolved in 10 mL of DI water in the Teflon-lined stainless autoclave. Subsequently, the Ni-Co (OH)_2_@NF precursor was immersed in the solution and maintained at 90 °C for 12 h. Finally, the obtained NiCo_2_S_4_@NF was washed three times with DI water and ethanol.

*Synthesis of UiO-66 (Hf):* UiO-66 (Hf) was synthesized following a modified solvothermal method.^[2]^ First, in a 100 mL Teflon-lined stainless-steel autoclave, HfCl_4_ (0.343 mmol) and benzoic acid (3.43 mmol) were dispersed in 20 mL of N, N-dimethylformamide (DMF). Subsequently, 2-aminoterephthalic acid (0.343 mmol) was introduced into the mixture and ultrasonicated for 3 min to ensure complete dispersion. 0.025 mL DI water was then added to the solution. The mixture solution was heated at 120 °C for 24 h. After cooling to room temperature, the resultant precipitates were collected by centrifugation. The product was purified by washing twice with DMF and DI water, then dried under vacuum.

*Cell culture:* Hala cells were cultured in Dulbecco’s Modified Eagle’s medium (DMEM) (Gibco, USA), supplemented with 10% (v/v) fetal bovine serum (FBS, Gibco, USA) and 100 U mL^-1^ penicillin/streptomycin (Gibco, USA). Cells were maintained in a humidified incubator at 37 °C with 5% CO_2_.

*Peroxidase-like activity detection:* The peroxidase-like activity of NiCo_2_S_4_@UiO-66 was assessed using chromogenic substrate TMB.^[3, 4]^ Briefly, The TMB (100uL, 2mM final concentration) solution and NiCo_2_S_4_@UiO-66 (2mg mL^-1^ final concentration) solution were added to 1.8 mL of acetate buffer (100 mM, pH = 4.0). The mixture was then subjected to US treatment (0.5 W cm^-2^, 50% cycle, 10 min duration, 1 MHz) for 2 min. Subsequently, 1 mL of H_2_SO_4_ (0.1 M) was added to quench the reaction and converted blue oxTMB into yellow product with a maximum absorbance at 450 nm. UV-vis spectra of the mixture were recorded before and after the reaction to confirm the TMB oxidation.

*Determination of ROS generation:* ROS generation induced by US-activated NiCo_2_S_4_@UiO-66 was evaluated using nonfluorescent probe 2’,7’-dichlorofluorescein diacetate (DCFH-DA). In each well of a 96-well plate, Different concentrations of NiCo_2_S_4_@UiO-66 were mixed with an appropriate amount of DCFH-DA, resulting in a total mixture volume of 150 μL. Following US exposure (0.5 W cm^-2^, 50% cycle, 10 min duration), the fluorescence intensity of the generated DCF was measured using a microplate reader with excitation at 488 nm.

*Monitoring the generation of hydroxyl radical (·OH) and singlet oxygen (**^1^O_2_):* To assess whether the generated ROS species include ·OH and ^1^O_2_, we conducted similar fluorescence-based ROS detection assays, using p-phthalic acid (PTA) as a probe for ·OH and SOSG as a probe for ^1^O_2_.^[5, 6]^ PTA was virtually non-fluorescent but became brightly green fluorescent upon oxidation by ·OH. SOSG was also virtually non-fluorescent but became brightly green fluorescent upon oxidation by ^1^O_2_. Specifically, PTA powder was dissolved in a 0.2 M NaOH aqueous solution to achieve a PTA concentration (50 mM). The resulting PTA solution was then mixed with a NiCo_2_S_4_@UiO-66 dispersion in PBS (with an expected mass concentration of NiCo_2_S_4_@UiO-66) to a final volume of 200 μL and a final PTA concentration (500 μM), followed by treated with different groups. Briefly, SOSG solution was mixed with a NiCo_2_S_4_@UiO-66 dispersion in PBS (with an expected mass concentration of NiCo_2_S_4_@UiO-66) to a final volume of 200 μL and a final SOSG concentration (10 μM), followed by treated with different groups. The resulting mixture was then ultrasonicated (0.5 W cm^-2^, 10 min) with a medical ultrasonic instrument, followed by centrifugation (10,000 g, for 10 min) to collect nanoparticle-free supernatant for subsequent fluorescence emission spectrum measurement (SOSG: λex/λem = 504 nm/510-700 nm; PTA: (λex = 315 nm; λem = 320-600 nm) with a fluorimeter. The controls were SOSG and PTA solution treated similarly in PBS but without any nanoparticles.

*In vitro cytotoxicity of NiCo_2_S_4_@UiO-66:* Briefly, approximately 10^4^ HaLa cells were seeded into each well of a 96-well microplate and cultured in 10% FBS-supplemented DMEM at 37 °C for 12 h to reach 85% confluency. The Hala cells were treated with NiCo_2_S_4_@UiO-66 solution (0.1 mL in FBS-supplemented DMEM) for 4 h, followed by washing with PBS three times and replenishing with fresh FBS-supplemented DMEM. The treated cells were subsequently incubated at 37 °C for another 24 h, followed by cell viability measure with the CCK-8 by adding 10 μL CCK-8 into each well and then incubating at 37 °C for 1 h. Cell viability was quantified by measuring the optical density at 450 nm with a microplate reader (Varioskan, Thermo). All experiments were performed in triplicate, and the results were presented as averages of three independent trials.

*Animal Experiment:* BALB/c mice (4-5 weeks old, 18-20 g) were purchased from Jiesijie co., Ltd. (Shanghai, China) for in vivo experiments. All animal experiments were approved by the Animal Ethics Committee of East China Normal University (approval no. m20230907, Shanghai, China). All handling and surgical procedures followed the approved guidelines.^[7, 8]^ The mice were randomly divided into 9 groups, including healthy, control, US, NiCo_2_S_4_, NiCo_2_S_4_+US, UiO-66, UiO-66+US, NiCo_2_S_4_@UiO-66 and NiCo_2_S_4_@UiO-66+US. Following inhalation anesthesia, a round wound (5-6 mm diameter) was created on the back of each mouse. To induce bacterial infection, the wounds were inoculated with the pre-cultured *MRSA* suspension (1 × 10^8^ CFU mL^-1^). Wound treatments for different experimental groups commenced on day 2 post-infection. Wound areas were monitored and photographed every other day throughout the study. After 17 days of treatment, mice were humanely euthanized, and skin tissues from the wound sites were collected for subsequent histological analyses. For histological analysis, the wound tissues (n = 3) were fixed in 10% neutral buffered formalin, processed routinely into paraffin, sectioned, stained for H&E, Masson and IF analysis.

**2. Characterization of UiO-66 (Hf).**

**Figure S1.** (A) Particle size distribution of UiO-66 nanoparticles. (B) TEM images of UiO-66 nanoparticles. (C) Elemental analysis of UiO-66 nanoparticles. (D) EDS elemental distribution map of UiO-66.

**3. Energy dispersive X-ray spectroscopy of NiCo_2_S_4_.**

**Figure S2.** EDS elemental distribution map of NiCo_2_S_4_.

**4. Zeta potential of NiCo_2_S_4_@UiO-66**

**Figure S3.** Zeta potential of NiCo_2_S_4_@UiO-66.

**5. The reproducibility and structural stability of NiCo_2_S_4_@UiO-66**

**Figure S4.** (A) Morphological characteristics of NiCo_2_S_4_@UiO-66 among different batches. The top row shows SEM images, and the bottom as TEM images. (B) Zeta potential between different batches. (C) Comparison of UiO-66 loading content (Hf element) in the different samples and batches using ICP-OES. (D) Morphological features of NiCo_2_S_4_@UiO-66 dispersed in PBS at different time (1d, 10d and 15d). (E) Morphological features of NiCo_2_S_4_@UiO-66 before and after US treatment.

**6. Ultraviolet photoelectron spectroscopy of NiCo_2_S_4_@UiO-66.**

**Figure S5.** VB-XPS were used to measure the position of valence band of (A) UIO-66 and (B)

NiCo_2_S_4_@UiO-66.

**7. Non-specific ultrasound effects on the catalytic activity.**

**Figure S6.** (A) Absorption spectra of TMB as a substrate different treatment groups in the presence of H_2_O_2_. (B) Fluorescence spectra in different systems using the PTA probe in the presence of H_2_O_2_.

**8. Kinetic parameters of POD-like activity.**

**Figure S7.** Time-dependent absorbance changes of oxTMB in (A) NiCo_2_S_4_@UiO-66 and (B) NiCo_2_S_4_@UiO-66+US solutions with different H_2_O_2_ concentrations.

**9. Comparison of POD-like activities** **among different NiCo_2_S_4_ composite nanozymes.**

**Figure S8.** Comparison of POD-like activities among different NiCo_2_S_4_ composite nanozymes.

**10. The potential thermal effects or tissue safety under US irradiation.**

**Figure S9.** (A) Thermal images of the NiCo_2_S_4_@UiO-66 solution at different times under a US power of 0.5 W cm^-2^. (B) Thermal images of the dorsal skin of mice with or without US treatment.

**11.** **Anti-biofilm of** **NiCo_2_S_4_@UiO-66 nanocomposite.**

**Figure S10.** CLSM images showing the distribution of different treatment in *MRSA* biofilms, with green fluorescence representing bacterial biofilm.

**12. Statistical analysis of NiCo_2_S_4_@UiO-66 anti-Biofilm indicators**

**Figure S11.** Biofilm thickness, cell counts (in Log CFU mL^-1^) and biofilm biomass of the US triggered by NiCo_2_S_4_@UiO-66 to treat biofilm (n = 3 independent replicates, mean ± SD).

**13. The evaluation of probe specificity in NiCo_2_S_4_@UiO-66 system.**

**Figure S12.** (A) Fluorescence spectra of the SOSG probe used for ^1^O_2_ detection in different treatment groups. (B) Fluorescence spectra of the HPF probe used for ·OH detection in the presence of H_2_O_2_ in different treatment groups.

**14. Evaluation of NiCo_2_S_4_@UiO-66 biosafety *in vitro*.**

**Figure S13.** (A) Schematic diagram of in vitro cytotoxicity experiment of HeLa/L929 cell. (B) Cell viability after treatment with different concentrations of NiCo_2_S_4_@UiO-66. (C) Live/dead fluorescence images of cells treated with different NiCo_2_S_4_@UiO-66. Scale bar = 20 μm. (D) Without or (E) with US irradiation, cell viability of L929 cells after treatment with different concentrations of NiCo_2_S_4_@UiO-66. (F) Without or (G) with US irradiation, the LDH release from L929 cells treated with different concentrations of NiCo_2_S_4_@UiO-66. (H) Cell viability of L929 cells following co-culture with NiCo_2_S_4_@UiO-66 at different time. n = 3, mean ± SD. Statistical analysis was carried out with a one-way ANOVA with Tukey’s multiple-comparison test. (**P* < 0.05, ***P* < 0.01, n.s. represented no significance).

**15. Intrinsic biosafety of** **NiCo_2_S_4_@UiO-66.**

**Figure S14.** (A) Schematic illustration of the schedule for evaluating the biosafety of treatment on NiCo_2_S_4_@UiO-66 in BALB/c mice. (B) Mice body weights over 7 days after administration of NiCo_2_S_4_@UiO-66. (C) Serum levels of IL-6, TNF-α and IL-1β at 24 h after treatment completion. (D) Serum levels of ALT, AST and ALB of liver function. (E) Serum levels of UA, BUN, and CREA of renal function. (F) Complete blood count (CBC) parameters. Blood levels of RBC, HGB, WBC, neutrophils, lymphocyte, monocytes, eosinophil and PLT. Control indicates treatment with PBS alone. n = 5, Mean ± SD. P values were calculated using two-sided Student’s *t* test, n.s.: *P* > 0.05. (G) H&E staining of major tissues and Masson staining of the wounds tissue collected from mice in the PBS and the NiCo_2_S_4_@UiO-66 groups.

**16. Serum pro-inflammatory cytokine level testing.**

**Figure S15.** Serum levels of (A) interleukin-6 (IL-6), (B) tumor necrosis factor-α (TNF-α), and (C) interleukin-1 beta (IL-1β) in *MRSA* infection-bearing mouse models after differing treatments. Bar heights are reported as the average ± standard deviation (n = 5 biologically independent mice in one trial). Statistical analysis was carried out with a one-way ANOVA with Tukey’s multiple-comparison test.

**17. Biosafety of the sonodynamic therapy mediated by NiCo_2_S_4_.**

**Figure S16.** Serum levels of (A) alanine aminotransferase (ALT), (B) aspartate aminotransferase (AST), (C) Albumin (ALB), (D) creatinine (CREA), (E) urine acid (UA) and (F) blood urea nitrogen (BUN) (n = 5 biologically independent mice in one trial). Statistical analysis was carried out with a one-way ANOVA with Tukey’s multiple-comparison test.

**18. H&E-stained organs.**

**Figure S17.** Microscopy images of hematoxylin and eosin (H&E)-stained organs collected from mice in the treatment group.

**References**

1. T. Li, B. Wang, Y. Cao, et al., “Energy-saving hydrogen production by seawater electrolysis coupling tip-enhanced electric field promoted electrocatalytic sulfion oxidation.” *Nature Communications* 15 (2024), 6173.

2. C. Zhang, D. Lei, C. Xie, X. Hang, C. He, and H.L. Jiang, “Piezo-photocatalysis over metal–organic frameworks: Promoting photocatalytic activity by piezoelectric effect.” *Advanced Materials* 33 (2021), 2106308.

3. Y. Cui, N. Zhao, S. Wang, H. Yan, and D. Han, “Colorimetric detection of tetracycline by enhancing peroxidase-like activity of polymerized MnO_2_@ZIF-67 nanocomposites.” *Biosensors and Bioelectronics* 287 (2025), 117726.

4. H. Li, M. Sun, H. Gu, et al., “Peroxidase-like fecozn triple-atom catalyst-based electronic tongue for colorimetric discrimination of food preservatives.” *Small* 19 (2023), 2207036.

5. F. Gao, T. Shao, Y. Yu, Y. Xiong, and L. Yang, “Surface-bound reactive oxygen species generating nanozymes for selective antibacterial action.” *Nature Communications* 12 (2021), 745.

6. T. Liu, S. Chai, M. Li, et al., “A nanoparticle-based sonodynamic therapy reduces helicobacter pylori infection in mouse without disrupting gut microbiota.” *Nature Communications* 15 (2024), 844.

7. G. Fang, Q. Dong, X. Shen, et al., “Modulation of bacterial iron homeostasis to enhance cuproptosis-like death for the treatment of infected diabetic wound.” *ACS Nano* 19 (2025): 15578-15595.

8. Y. Jin, Y. Lu, X. Jiang, et al., “Accelerated infected wound healing by probiotic-based living microneedles with long-acting antibacterial effect.” *Bioactive Materials* 38 (2024): 292-304.

9. J. Xia, C. Yang, M. Wang, et al., “Engineering sulfur vacancies in NiCo_2_S_4_ flower-like structure to achieve exceptional peroxidase activity for advanced colorimetric biomedical diagnostics.” *Journal of Alloys and Compounds* 1030 (2025), 180924.

10. Y. He, N. Li, W. Li, et al., “5,10,15,20-tetrakis (4-carboxylphenyl) porphyrin functionalized NiCo_2_S_4_ yolk-shell nanospheres: Excellent peroxidase-like activity, catalytic mechanism and fast cascade colorimetric biosensor for cholesterol.” *Sensors and Actuators, B: Chemical* 326 (2021), 128850.

11. H. Ye, Y. Ding, T. Liu, et al., “Colorimetric assay based on NiCo_2_S_4_@N,S-rGO nanozyme for sensitive detection of H_2_O_2_ and glucose in serum and urine samples.” *RSC Advance* 12 (2022): 20838-20849.

12. M. Lian, M. Liu, X. Zhang, et al., “Template-regulated bimetallic sulfide nanozymes with high specificity and activity for visual colorimetric detection of cellular H_2_O_2_.” *ACS Applied Materials & Interfaces* 13 (2021): 53599-53609.

13. Z. Huang, W. He, H. Shen, et al., “NiCo_2_S_4_ microflowers as peroxidase mimic: A multi-functional platform for colorimetric detection of glucose and evaluation of antioxidant behavior.” *Talanta* 230 (2021), 122337.

14. X. Wang, M. Chen, and L. Zhao. “Development of a colorimetric sensing assay for ascorbic acid and sarcosine utilizing the dual-class enzyme activity of Fe_3_O_4_@SiO_2_@NiCo_2_S_4_.” *Chemical Engineering Journal* 468 (2023), 143612.
